# Associations of Lifestyle and Anthropometric Factors With the Risk of Herpes Zoster: A Nationwide Population-Based Cohort Study

**DOI:** 10.1093/aje/kwab027

**Published:** 2021-02-11

**Authors:** Sigrun A J Schmidt, Henrik Toft Sørensen, Sinéad M Langan, Mogens Vestergaard

**Keywords:** alcohol consumption, body mass index, exercise, herpes zoster, smoking, cohort study

## Abstract

The role of lifestyle in development of herpes zoster remains unclear. We examined whether smoking status, alcohol consumption, body mass index, or physical activity were associated with zoster risk. We followed a population-based cohort of 101,894 respondents to the 2010 Danish National Health Survey (baseline, May 1, 2010) until zoster diagnosis, death, emigration, or July 1, 2014, whichever occurred first. We computed hazard ratios for zoster associated with each exposure, using Cox regression with age as the time scale and adjusting for potential confounders. Compared with never smokers, hazards for zoster were increased in former smokers (1.17, 95% confidence interval (CI): 1.06, 1.30), but not in current smokers (1.00, 95% CI: 0.89, 1.13). Compared with low-risk alcohol consumption, neither intermediate-risk (0.95, 95% CI: 0.84, 1.07) nor high-risk alcohol consumption (0.99, 95% CI: 0.85, 1.15) was associated with zoster. We also found no increased hazard associated with weekly binge drinking versus not (0.93, 95% CI: 0.77, 1.11). Risk of zoster varied little by body mass index (referent = normal weight) and physical activity levels (referent = light level), with hazard ratios between 0.96 and 1.08. We observed no dose-response association between the exposures and zoster. The examined lifestyle and anthropometric factors thus were not risk factors for zoster.

## Abbreviations

BMI: body mass index
CI: confidence interval
HR: hazard ratio

Herpes zoster (zoster, or shingles) is characterized as a unilateral vesicular exanthema with acute neuritis (1). It is caused by varicella-zoster virus, which is acquired during primary infection (chickenpox), when it establishes latency in sensory ganglia, making the person at risk for development of zoster later in life if cell-mediated immunity fails to suppress viral activity. Although approximately one-third of the population contracts zoster during the life course (1), there are few well-established risk factors for reactivation besides female sex, increasing age, and immunosuppressive treatments and diseases (e.g.*,* glucocorticoids, chemotherapy, and hematological cancer( (1, 2).

Cigarette smoking (3), excessive alcohol intake (4), overweight (5), and vigorous physical activity (6) may be immunosuppressive, according to results of earlier studies. Because reduced cell-mediated immunity is a risk factor for reactivation of varicella-zoster virus, such habits, in turn, could trigger zoster. However, results from epidemiologic studies have been inconsistent. A decreased relative risk of zoster among smokers (particularly current smokers) have been reported in several studies (7–12), whereas no association was found in others (13–18). In a single, small case–control study, researchers reported an association with alcohol consumption (odds ratio = 3.8) (16); otherwise, no substantial associations with zoster have been observed for alcohol consumption (7, 8, 14, 16, 17), body mass index (BMI) (7, 8, 14, 17, 19), or physical activity (8). Interpretation of existing evidence is hampered by crude categorization of exposures; particularly, there is a lack of dose–response analyses. Furthermore, studies were limited by potential confounding and small sample sizes.

Zoster can lead to serious acute and chronic complications, including postherpetic neuralgia, a debilitating condition causing prolonged pain, decreased quality of life, and possible loss of functional capacity (1). Given the high lifetime prevalence and these potential individual and societal implications of zoster, identification of modifiable risk factors is a priority. We conducted a large, population-based cohort study in Denmark to examine whether lifestyle and anthropometric factors, including smoking habits, alcohol consumption, BMI, and physical activity, were associated with risk of zoster, taking important confounding factors into account and evaluating possible dose–response associations.

## METHODS

### Study population

The study cohort comprised participants in the nationwide Danish National Health Survey, conducted in 2010 (20) among Danish inhabitants aged at least 16 years on January 1, 2010. Participants were sampled through the Civil Registration System’s database of Danish residents (21). Of 298,850 persons invited, 60% responded to the Survey, which included questions about sociodemographic characteristics, health-related quality of life, health behavior, morbidity, consequences of illness, and social relations (20).

We had access to selected Survey variables for the population at least 25 years old on January 1, 2010. Questionnaire collection was completed on May 1, 2010, which we defined as baseline. We excluded persons who completed the questionnaire but died or emigrated before completion of collection. We linked the survey data to records of hospital diagnoses and treatment in the Danish National Patient Registry (22) and the Danish Central Psychiatric Research Registry (23), as well as to prescriptions recorded in the Danish National Prescription Registry (24), demographic and vital status data in the Civil Registration System (21), and education level recorded in the Population Education Registry (25). Additional information on the Survey and the registries used in the study is provided in the Web Appendix (available at https://doi.org/10.1093/aje/kwaa027).

### Exposures

We used information from the Survey to study several aspects of lifestyle and their association with subsequent risk of zoster. Table 1 lists definitions and working hypotheses for the exposures of interest, including how they may affect the immune system and thus increase zoster risk. Detailed code lists for all study variables are presented in Web Table 1.

**Table 1:** Definitions and A Priori Working Hypotheses for Exposures of Interest in the Study on Lifestyle and Anthropometric Factors and Risk of Herpes Zoster (*n* = 101,894), 2010 Danish National Health Survey

**Variable**	**Definition**	**A Priori Working Hypothesis** ^**a**^
Smoking status	We categorized persons as never smokers (referent), former smokers, or current smokers (daily or nondaily).	Cigarette smoke affects both innate and adaptive immunity (3). Although cigarette smoking results in leukocytosis in humans, the function of circulating cells is reduced. This effect is thought to be mediated by nicotine and/or tar. We hypothesized that current smokers are at increased risk of zoster, whereas former smokers have no or slightly increased risk.
Average daily tobacco consumption	Among current daily smokers of tobacco, we calculated total daily consumption in grams, using the following rule: 1 cigarette = 1 g, 1 cigar = 4.5 g, 1 cheroot = 3 g, and 1 pipe stop = 3 g of tobacco. We grouped total consumption by 5-g increments: 1–5, 6–10, 11–15, 16–20, 21–25, 26–30, and ≥30 g. In a restricted cubic splines model, we included daily consumption as a continuous variable, rounding to the nearest full integer and capped at ≥40 g/day. Never smokers constituted the reference group.	The risk of zoster increases with increasing daily tobacco consumption, possibly reaching a plateau after a certain level of daily consumption.
Weekly alcohol consumption	On the basis of the number of standardized alcohol units consumed weekly, we categorized drinking as at low risk (≤ 7 units for women; ≤ 14 units for men), intermediate risk (8–14 units for women; 15–21 units for men), or high risk (≥ 15 units for women; ≥ 22 units for men). We included nondrinkers in the low-risk group (reference group). In a restricted cubic splines model, we included the number of units consumed weekly as a continuous variable, capped at ≥80 units, and with consumption of 0 units as the referent.	Alcohol has been associated with increased T-cell apoptosis, reduced interleukin-17 production, lymphopenia in blood and tissues, and reduced activity of antigen-presenting cells (4). We hypothesized that risk of zoster increases with an increasing number of units of alcohol consumed per week.
Binge drinking	We defined binge drinkers as those who reported daily, almost daily, or weekly intake of ≥ 5 units of alcohol on a single occasion. Those reporting binge drinking monthly or less constituted the reference group.	We hypothesized that binge drinking is associated with increased risk of zoster, similar to that for weekly alcohol consumption.
BMI category	We categorized BMI^b^ according to the World Health Organization definition: underweight (BMI < 18.5); normal weight (BMI 18.5–24.9; referent); overweight (BMI 25–29.9); or obese (BMI ≥ 30). In a restricted cubic splines model, we included BMI as a continuous variable, rounding to the nearest full integer, capped at ≥60, with BMI = 22 as the referent.	Obesity is characterized by low-grade chronic inflammation and also has been linked to functional immune deficiency, as evidenced by impaired lymphocyte proliferation to antigen stimulation, reduced T-cell diversity, enhanced aging of the thymus, an increased risk of some infections among obese persons, and possibly reduced immunogenicity of vaccinations (5). We hypothesized that there is a J- or U-shaped relation between BMI and risk of zoster, with an increased risk among underweight persons (related to potential undernutrition) and among overweight and obese persons.
Physical activity	We categorized physical activity as sedentary (reading, watching television, or other sedentary activity most of the time); light (walking, cycling, or other light exercise ≥ 4 hour/week, including Sunday excursions, light gardening, and cycling or walking to work; referent); or moderate/vigorous (heavy exercise and competitive sports regularly and several times a week; or recreational sports, heavy gardening, or a similar activity ≥ 4 hours weekly).	Exercise influences natural immunity, T-cell and B-cell functions, and cytokine responses, through hemodynamic and endocrine changes (6). The mechanisms are complicated (e.g., longer periods of intensified training in athletes may decrease T-cell functionality, whereas this effect is not observed for persons with a previously sedentary lifestyle. We hypothesized that there is a J- or U-shaped relationship between physical activity and risk of zoster, with lowest risk among those performing light and moderate physical activity and highest risk among those participating in vigorous activity.

Abbreviation: BMI, body mass index

^a^ Describes how factors may affect the immune system and thus increase the potential risk of zoster.

^b^ Weight (kg)/height (m)^2^.

### Outcome

We followed persons from baseline until zoster diagnosis, death, emigration, or July 1, 2014, whichever came first. We used a validated algorithm (26) to identify zoster as either (1) a first-time inpatient, outpatient clinic (ambulatory), or emergency room primary or secondary hospital registry diagnosis of this condition recorded in the Danish National Patient Registry; or (2) a first-time prescription for 35 pills of acyclovir at an 800-mg dose or for valacyclovir or famciclovir at a 500-mg tablet dose (i.e., prescriptions likely to represent treatment for zoster). To avoid including prevalent zoster cases and misclassification of treatment of herpes simplex, we excluded persons who were not older than 40 years at baseline, persons who had a previous diagnosis of zoster or postherpetic neuralgia, and persons with a previous prescription for acyclovir, valacyclovir, or famciclovir at any dose (26). We considered the first recorded date of the qualifying hospital contact or prescription to be the diagnosis date.

In Northern Europe, chickenpox is considered a childhood disease; it is estimated that more than 98% of adult Danes are seropositive to varicella-zoster virus and thereby at risk of zoster (27, 28). Varicella vaccination is not part of the routine childhood vaccination schedule in Denmark and no zoster vaccine was marketed in Denmark during the study period.

### Covariables

On the basis of a directed acyclic graph for our study (Web Figure 1), we identified potential confounders recorded in our data sources before or at baseline (Web Table 1). We thus included age, sex, and various immunosuppressive treatments, and chronic conditions (2, 26), including rheumatoid arthritis, systemic or subacute lupus erythematosus, inflammatory bowel disease, chronic kidney disease, asthma, chronic obstructive pulmonary disease, use of inhaled glucocorticoids, diabetes mellitus, hospital-diagnosed mood disorders, and severe immunosuppression (e.g., human immunodeficiency virus infection, hematopoietic stem cell transplantation, other cellular immune deficiency, leukemia, lymphoma, myeloma, use of oral glucocorticoids or other immunosuppressant drugs). We also identified the highest level of education achieved by each person (as a measure of socioeconomic status, which could affect lifestyle habits and health-seeking behavior (29)) and country of origin as proxy for ethnicity (associated with zoster (29) and potentially linked to lifestyle through socioeconomic or other factors).

### Statistical analyses

We characterized the study cohort at baseline, both overall and in exposure categories. For each exposure level, we computed the number of events, follow-up time, and incidence rate of zoster. We used Cox proportional hazards regression (30) stratified by 5-year birth cohorts to compute hazard ratios with 95% confidence intervals associating each exposure with zoster. We used age as the time scale, because of the strong association between age and zoster and because it did not require that we specify the nature of the association. We explored the impact of covariable adjustment by fitting sequential multivariable models. Model 1 was adjusted for sex. Model 2 additionally included immune-related diseases and immunosuppressive drugs. Model 3 also included country of origin and education level. Finally, using model 4, we explored the effects of adding other relevant exposures of interest: smoking habits were adjusted additionally for weekly alcohol consumption, BMI, and physical activity; variables for alcohol consumption were adjusted for smoking status, BMI, and physical activity; and BMI and physical activity were adjusted for smoking status and weekly alcohol consumption. We examined associations with daily tobacco consumption, weekly alcohol consumption, and BMI—both as categorical variables and as continuous variables in restricted cubic splines models. We used the variation inflation factor from a regression including variables from model 4 to examine whether collinearity occurred. We found no evidence of collinearity (individual variance inflation factors were ≤ 1.58; mean values were ≤ 1.12).

We used stratified analyses to examine the possibility of effect-measure modification by age group at baseline and sex. For example, some lifestyle factors could have an impact on immune function and accelerate its decline in elderly persons who already have a weaker immune system. Furthermore, because we expected high BMI and a sedentary lifestyle to be closely linked, and possibly to have a multiplicative effect, we examined associations for each BMI category in strata of physical activity and vice versa. In a sensitivity analysis, we examined whether adjustment for additional socioeconomic variables (namely, working status and civil status) affected our main results.

We accounted statistically for survey design and differential nonresponse in all analyses by incorporating calibrated postsurvey weights computed by Statistics Denmark. These weights were based on selected characteristics identified through linkage to other data sources (31). We assessed the proportional hazards assumption for each exposure by visual inspection of log–log plots and found no substantial deviations.

We performed all analyses using Stata, version 15. The study was approved by the Danish Data Protection Agency (record no. 2013-41-1719). Approval by an ethics review board and informed consent were not required.

### Missing data

Some information was missing for self-reported variables. We considered data unlikely to be “missing at random,” because reporting of lifestyle variables could be related to the true value (e.g., missing data on alcohol consumption among heavy drinkers). Although the missingness mechanism cannot be identified with observational data, these data can help to frame plausible assumptions (32). We used 3 strategies to investigate missing information: 1) we examined the distribution of missing data, 2) summarized data for fully observed (or near-fully observed) variables by missingness pattern, and 3) computed odds ratios using logistic regression with an indicator for missingness as the dependent variable (32). We included variables from model 4. Age was entered as a categorical variable. Furthermore, we compared hazard ratios for zoster associated with study covariables (where available) among participants with complete data with that among survey nonresponders. For comparability, this analysis included nonresponders who fulfilled study eligibility criteria (i.e., age > 40 years and no record suggesting previous zoster, postherpetic neuralgia, or antiviral treatment), and we did not use survey information for defining covariables (e.g., rheumatoid arthritis was identified based on hospital-diagnoses only).

Frequency of missing data on exposures varied between 2.1% for education to 5.1% for alcohol consumption (Web Table 2). Approximately 85% of persons had complete data on all variables. Descriptive analyses (Web Table 3) and multivariable logistic regression (Web Table 4) showed that missingness was not associated with zoster but was with several confounders, including older age, female sex, shorter education, several chronic diseases, “other” country of origin, and, for individual exposures, with lower alcohol consumption, lower BMI, and a more sedentary lifestyle. This pattern is consistent with missingness that is conditionally independent of an outcome. The pattern also fits the study’s prospective design (32), in which the outcome was unknown at the time of the Survey and willingness to respond to a question was thus unlikely to be associated with later risk of zoster. We used complete-case regression analysis for our analysis because this approach provides asymptotically unbiased estimates of the exposure association in situations characterized by this type of missingness (32). We expected only minor loss of precision, given the modest prevalence of missing data. The hazard ratios for the association between covariables and zoster were comparable for the final study population and for nonresponders (Web Figure 2).

**Figure 1:**
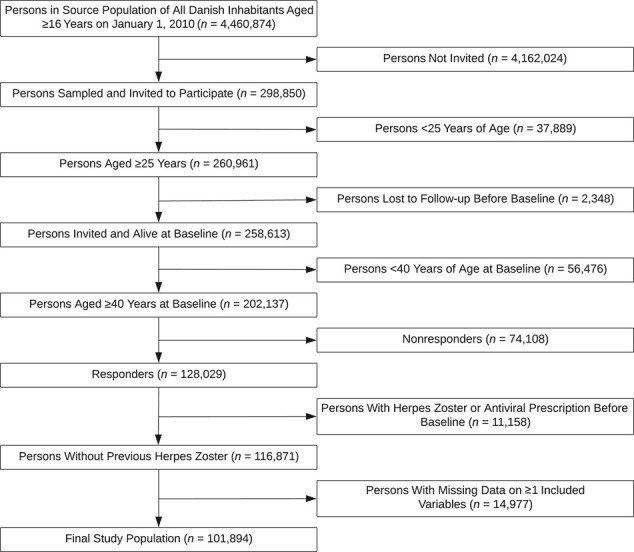
Flowchart for the study on lifestyle and anthropometric factors and risk of herpes zoster, based on data from the 2010 Danish National Health Survey.

## RESULTS

The study flowchart is presented in Figure 1. In total, 101,894 persons fulfilled the study’s eligibility criteria. Mean age was 58 years (standard error, 0.044) and 49.2% of participants were women (Table 2). The majority were Danish (92.9%) and had an intermediate level of education (49.6%). Baseline characteristics by levels of exposures are presented in Web Tables 5–8. Exposure categories representing the healthiest lifestyle choices (e.g., never smokers and normal-weight persons) tended to have higher proportions of women (except for the most physically active), certain chronic diseases, lower level of education, and generally healthy lifestyle.

**Figure 2:**
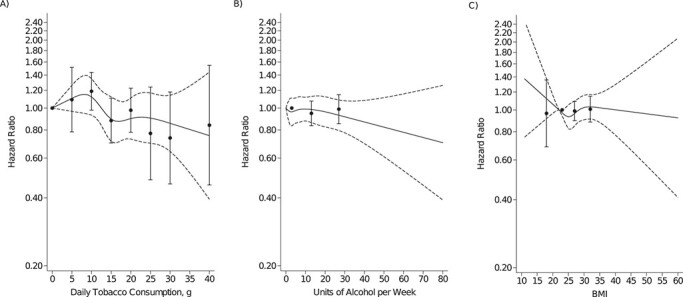
Hazard ratios (95% confidence intervals) for herpes zoster associated with A) daily tobacco consumption, B) weekly alcohol consumption, and C) body mass index (BMI; calculated as weight (kg)/height (m)^2^), entered as restricted cubic spline models (solid and dashed lines) and as categorical variables (circles with error bars), 2010 Danish National Health Survey.

In total, 2,231 persons were diagnosed with zoster during 357,768 person-years of follow-up (mean = 4.0 years; standard error, 0.0023), yielding an incidence rate of 6.2 per 1,000 person-years. Compared with never smokers, former smokers had increased hazards for zoster (fully adjusted hazard ratio (HR) = 1.17, 95% confidence interval (CI): 1.06, 1.2′30), but not current smokers (HR = 1.00, 95% CI: 0.89, 1.13). Among current daily smokers who reported quantity of tobacco consumption (*n* = 21,310; 98.5%), we found no dose–response relation with risk of zoster (referent = never smokers), although sample size was small for the upper strata in which a decreased hazard ratio could not be excluded. Compared with low-risk alcohol consumption, neither intermediate-risk (HR = 0.95, 95% CI: 0.84, 1.07) nor high-risk (HR = 0.99, 95% CI: 0.85, 1.15) consumption was associated with an increased hazard of zoster. Similarly, relative risk was not increased in respondents who reported weekly binge drinking versus those who did not (HR = 0.93, 95% CI: 0.77, 1.11). We also found no variation in hazard of zoster by levels of BMI (referent = normal weight) or physical activity (referent = light lifestyle), with hazard ratios ranging between 0.98 and 1.08. Analyses in which daily tobacco consumption, weekly alcohol consumption, and BMI were entered as cubic splines supported the results for categorical exposure definitions (Figure 2).

**Table 2:** Baseline Characteristics of the Study Cohort (*n* = 101,894), 2010 Danish National Health Survey^a^

**Variable**	**No.**	**%**
Age group, years		
40–49	27,382	30.8
50–59	23,703	26.7
60–69	22,351	25.1
70–79	10,886	12.2
≥80	4,584	5.2
Sex		
Men	45,189	50.8
Women	43,717	49.2
Immunosuppressive and chronic diseases		
Rheumatoid arthritis	6,720	7.6
Systemic lupus erythematosus	90	0.1
Inflammatory bowel disease	1,062	1.2
Chronic kidney disease	530	0.6
Asthma	2,558	2.9
Chronic obstructive pulmonary disease	6,014	6.8
Inhaled corticosteroids	3,569	4.0
Diabetes	6,123	6.9
Mood disorder	4,731	5.3
Severe immunosuppression	2,491	2.8
Human immunodeficiency virus	56	0.1
Other immunosuppressive disease	31	0
Leukemia	137	0.2
Lymphoma	316	0.4
Myeloma	35	0
Oral corticosteroids	1,161	1.3
Hematopoietic stem cell transplant	62	0.1
Other immunosuppressants	985	1.1
Highest achieved level of education^b^		
Short	23,747	26.7
Intermediate	44,135	49.6
High	21,023	23.6
Country of origin^c^		
Danish	82,625	92.9
Other Western	3,226	3.6
Non-Western	3,054	3.4
Smoking status		
Never	35,774	40.2
Former	30,809	34.7
Current	22,322	25.1
Alcohol consumption^d^		
Low-risk	67,645	76.1
Intermediate-risk	12,099	13.6
High-risk	9,161	10.3
Binge drinking weekly or more		
No	80,634	90.7
Yes	8,272	9.3
Body mass index category		
Underweight	1,386	1.6
Normal	39,593	44.5
Overweight	34,050	38.3
Obese	13,877	15.6
Physical activity		
Sedentary	13,710	15.4
Light	55,124	62.0
Moderate	18,494	20.8
Vigorous	1,577	1.8

^a^ In all analyses, we included calibrated weights to statistically account for survey design and differential nonresponse. Thus, the numbers may not add up to the total.

^b^ Categorized as short (< 10 years), intermediate (10–15 years), and high education (> 15 years), according to the United Nations Educational, Scientific and Cultural Organization’s classification

^c^ Defined using an algorithm that combines data on citizenship, place of birth, and parents’ place of birth, as recorded in the Civil Registration System.

^d^ Categorized based on number of standardized alcohol units consumed weekly: low risk (≤ 7 for women; ≤ 14 for men) intermediate risk (8–14 for women; 15–21 for men); or high risk (≥ 15 for women; ≥ 22 for men).

**Table 3:** Association Between Lifestyle and Anthropometric Factors and Herpes Zoster (*n* = 101,894), 2010 Danish National Health Survey^a^

**Lifestyle and Anthropometric Factors**	**Events**	**Person-Years**	**Rate (Per 1,000 Persons)**	**Model 1**		**Model 2**		**Model 3**		**Model 4**	
				**HR** ^**b**^	**95% CI**	**HR** ^**b**^	**95% CI**	**HR** ^**b**^	**95% CI**	**HR** ^**b**^	**95% CI**
Smoking status											
Never	834	145,274	5.74	1.00	(Referent)	1.00	(Referent)	1.00	(Referent)	1.00	(Referent)
Former	892	123,196	7.24	1.20	1.09, 1.33	1.17	1.06, 1.29	1.17	1.06, 1.29	1.17	1.06, 1.30
Current	505	89,298	5.66	1.04	0.92, 1.17	1.00	0.89, 1.13	1.00	0.89, 1.13	1.00	0.89, 1.13
Tobacco consumption, g/day^c^											
1–5	42	6,156	6.84	1.12	0.80, 1.56	1.09	0.78, 1.51	1.08	0.78, 1.50	1.09	0.78, 1.51
6–10	142	19,234	7.40	1.24	1.02, 1.49	1.20	0.99, 1.45	1.18	0.98, 1.43	1.19	0.98, 1.44
11–15	96	18,776	5.09	0.93	0.74, 1.17	0.89	0.71, 1.11	0.87	0.69, 1.09	0.88	0.70, 1.11
16–20	116	21,622	5.38	1.05	0.85, 1.30	0.99	0.80, 1.23	0.97	0.78, 1.21	0.98	0.78, 1.23
21–25	21	5,133	4.07	0.83	0.52, 1.32	0.77	0.48, 1.24	0.76	0.47, 1.21	0.77	0.48, 1.24
26–30	20	5,103	3.89	0.79	0.50, 1.26	0.73	0.46, 1.17	0.72	0.45, 1.15	0.74	0.46, 1.18
≥30	15	3,411	4.45	0.93	0.50, 1.70	0.84	0.46, 1.56	0.83	0.45, 1.53	0.84	0.46, 1.54
Alcohol consumption risk^d^											
Low	1,697	272,241	6.23	1.00	(Referent)	1.00	(Referent)	1.00	(Referent)	1.00	(Referent)
Intermediate	309	48,926	6.32	0.95	0.83, 1.07	0.96	0.85, 1.09	0.96	0.84, 1.08	0.95	0.84, 1.07
High	225	36,601	6.15	1.00	0.86, 1.15	1.00	0.86, 1.15	0.99	0.86, 1.15	0.99	0.85, 1.15
Binge drinking weekly or more											
No	2,072	324,431	6.39	1.00	(Referent)	1.00	(Referent)	1.00	(Referent)	1.00	(Referent)
Yes	160	33,338	4.79	0.94	0.78, 1.12	0.93	0.77, 1.11	0.92	0.77, 1.11	0.93	0.77, 1.11
Body mass index category											
Underweight	42	5,256	7.98	1.00	0.71, 1.41	0.95	0.67, 1.33	0.95	0.67, 1.33	0.96	0.69, 1.36
Normal	1,015	159,066	6.38	1.00	(Referent)	1.00	(Referent)	1.00	(Referent)	1.00	(Referent)
Overweight	825	137,547	6.00	0.99	0.90, 1.10	0.99	0.90, 1.10	1.00	0.91, 1.10	0.99	0.90, 1.09
Obese	349	55,899	6.25	1.04	0.91, 1.18	1.02	0.89, 1.16	1.02	0.89, 1.16	1.01	0.88, 1.15
Physical activity											
Sedentary	378	52,680	7.18	1.04	0.92, 1.18	0.99	0.87, 1.12	0.99	0.87, 1.12	0.99	0.87, 1.13
Light	1,429	223,098	6.41	1.00	(Referent)	1.00	(Referent)	1.00	(Referent)	1.00	(Referent)
Moderate	393	75,566	5.20	0.97	0.87, 1.09	0.99	0.88, 1.11	0.99	0.88, 1.11	0.98	0.88, 1.11
Vigorous	31	6,424	4.88	1.07	0.72, 1.59	1.08	0.73, 1.62	1.08	0.73, 1.62	1.08	0.72, 1.61

Abbreviations: CI, confidence interval; HR, hazard ratio

^a^ In all analyses, we included calibrated weights to statistically account for survey design and differential nonresponse. Thus, the numbers may not add up to the total.

^b^ Computed using Cox regression with age as underlying time scale and stratified by birth cohort. Model 1 was adjusted for sex. Model 2 was adjusted additionally for the immunosuppressive and chronic diseases listed in Table 2, and model 3 also included country of origin and education level. Model 4 additionally was adjusted for other lifestyle factors (i.e., smoking status was adjusted for weekly alcohol consumption, body mass index, and physical activity; weekly alcohol consumption and binge drinking were adjusted for smoking status, body mass index, and physical activity; and body mass index and physical activity were adjusted for smoking status and weekly alcohol consumption.

^c^ Among daily smokers compared with never smokers.

^d^ Categorized on the basis of the number of standardized alcohol units consumed weekly: low risk (≤ 7 for women; ≤ 14 for men) intermediate risk (8–14 for women; 15–21 for men); or high risk (≥ 15 for women; ≥ 22 for men).

We did not find evidence of effect modification by age, sex, physical activity (for BMI), and BMI (for physical activity), but analyses were limited by few events and, thus, overlapping CIs (Web Tables 9–11).

Additional adjustment for working status and civil status factors had no effect on estimates from the main analysis (Web Table 12). Hazard ratios for zoster associated with covariables in the fully adjusted models are provided in Web Table 12. In a post hoc analysis, we also adjusted results for new diagnoses (recorded within the year before baseline) of diseases included in the Charlson Comorbidity Index (33), because we suspected residual confounding by comorbidity of the results for former smokers; however, this had no substantial impact on results (Web Table 13).

## DISCUSSION

In this population-based cohort study, we found no associations of smoking, alcohol consumption, BMI, or level of physical activity with risk of zoster, except for a slightly increased hazard among former smokers compared with never smokers.

Authors of previous studies on smoking status and risk of zoster have reported conflicting results. One case–control study (7), 2 cohort studies using prospectively collected population-based data (8, 9), and 2 small case–control studies using self-reported data (10, 11) found a 7%–48% decreased relative risk of zoster among current smokers and either decreased (0.87 (9)) or slightly increased estimates of relative risk (1.06–1.27 (7, 8, 10, 11)) among former smokers. Similarly, in a US population-based cohort study with 4,162 participants, researchers reported a hazard ratio of 0.47 (95% CI: 0.25, 0.89) for current versus “other” smoking (12). Studies with less clear definitions of smoking (ever or unspecified) generally found no association with zoster (13–18). To our knowledge, only 1 study tried to evaluate dose–response: in a Japanese cohort study with 12,351 participants, the hazard ratios of zoster were 0.48 (95% CI: 0.27, 0.85) for participants who smoked 1–19 cigarettes daily and 0.56 (95% CI: 0.31, 1.00) for those who smoked more than 20 cigarettes daily, compared with never smokers (9). The increased risk of zoster among former smokers in our study partly agrees that reported in previous studies. It is possible that diagnosis of a chronic disease may both motivate smoking cessation and increase risk of zoster, but our post hoc analysis did not support strong confounding by recent diagnoses of chronic diseases included in the Charlson Comorbidity Index. Nevertheless, the lack of an association with current smoking and of a dose–response relation suggests this finding could be explained by noncausal mechanisms.

Among 3 small case–control studies (14, 16, 17), 1 reported an increased crude odds ratio (3.8, 95% CI: 1.15, 2.5) for the association between alcohol consumption (without further specification) and zoster. The validity of these studies was compromised by inaccurate exposure definitions, selection bias (e.g., due to missing data), recall bias, confounding (2 presented only crude data), and small sample sizes (between 44 and 250 cases). In a larger case–control study (n = 144,959 cases; n = 549,336 controls) based on routinely collected data in the United Kingdom, Forbes (7) found odds ratios for zoster close to unity among current drinkers (1.05, 99% CI: 1.02, 1.08) and former drinkers (1.11, 99% CI: 1.07, 1.15). A cohort study by Liu et al. (8) comprising participants in an Australian survey (The 45 and Up Study) was comparable to our study. Those authors also found no association between zoster and consumption of fewer than 7 alcohol units per week (HR = 1.02, 95% CI: 0.96, 1.08) or 7 or more alcohol units per week (HR = 1.04, 95% CI: 0.98, 1.10) compared with no consumption. By providing more detailed data on level of alcohol consumption, including binge drinking, our study provides reassuring consistency with the largely neutral findings of these well-controlled studies.

Our results also confirmed the null association between BMI and zoster reported in 5 previous studies (7, 8, 14, 17, 19). Three were small case–control studies with 44–250 cases that merely compared mean BMI values in persons with and without zoster (14, 17, 19). The other 2 large population-based studies (7, 8) compared BMI categories and reported relative risk estimates for zoster of 1.00–1.04 for overweight and obese persons compared with normal-weight or lower-weight persons.

A U-shaped association has been suggested for exercise, immune function, and risk of respiratory infections, with the lowest risk observed among persons with moderate exercise conditioning (6). We did not find such an association between physical activity and zoster. Only Liu et al. (8) examined this association, albeit with less detailed data. They similarly found no increase in the hazard of zoster for participants reporting vigorous physical activity once or more per week compared with less than once per week (0.98, 95% CI: 0.93, 1.03) (8).

A major strength of our study is our use of prospectively collected data from a tax-funded health care system with no copayment required by patients seeking help. Our study is also larger and has more detailed data on lifestyle factors, BMI, and important zoster risk factors, compared with most previous studies. Several potential weaknesses also must be considered. The response rate was 63% in our sample. However, we believe that selection bias was limited. First, loss to follow-up was minimal. Second, comparisons were made internally among participants. Third, we used survey weights to account for differential nonresponse. Fourth, associations between other study variables and zoster were largely independent of survey participation (Web Figure 2). Finally, selection bias due to missing data also seems unlikely, judging from patterns of missingness (Web Table 4) (32).

Self-reporting may have caused misclassification of exposures, but the study’s prospective design protected against differential recall bias. Nevertheless, in studies using survey data, there is a tendency for underreporting of characteristics such as BMI among overweight individuals (34) and smoking and alcohol consumption among frequent users (35). For exposure variables with more than 2 levels, such misreporting may result in bias toward the null for the lower and upper categories and away from the null for intermediate categories (36). However, our results did not indicate such patterns. Questionnaire data also present the risk of simultaneous misclassification of variables (e.g., misreports of both smoking and alcohol consumption) (36). Bias and residual confounding that stem from such dependent nondifferential misclassification are difficult to predict, but estimates remained consistent across our multivariable models. Finally, misclassification due to changes in lifestyle habits and BMI over time is possible, because we only had baseline data. For the same reason, some covariables may have acted both as confounders and mediators.

We supplemented hospital diagnoses of zoster with prescriptions for antiviral medications (positive predictive value = 87% (26)) to increase completeness. Although this approach is likely to identify most patients with zoster, it misses persons who never seek care or are not prescribed specific antivirals. Such misclassification may be differential if certain lifestyle habits are associated with health-seeking behavior and thus with an increased chance of being diagnosed and treated for zoster (ascertainment bias). However, associations of interest were neutral in our study and unaffected by adjustment for several variables potentially associated with health care contacts.

We adjusted for a wide range of zoster risk factors at baseline, using data from multiple sources to increase completeness. Nevertheless, residual confounding remains possible due to incomplete or crudely defined covariables or changes in status during follow-up. This may explain the increased risk found for former smokers.

Survey participation was associated with certain characteristics, such as sex and country of origin. However, the generalizability of our findings would be affected only if these factors modified study associations and if their prevalence differed in the target population (36, 37). We expect that our results could be generalized to populations similar to the Danish population. Unfortunately, we were unable to conduct informative stratified analyses examining the validity of extrapolation to other populations (e.g., those in which other ethnicities are predominant). Future studies could examine other aspects of lifestyle (e.g.*,* micronutrient intake and sleep habits) that could act as individual zoster risk factors, or researchers could modify associations examined in the current study.

In conclusion, we found no evidence of an association between lifestyle and anthropometric factors (i.e., smoking, alcohol consumption, BMI, and physical activity) and risk of zoster. The increased risk observed among former smokers may relate to poorer general health. The continued lack of known modifiable risk factors for zoster underscores the need for alternative preventive measures, such as vaccination.

## Supplementary Material

Web_Material_kwab027
