# Supplementary material for: Associations of Lifestyle and Anthropometric Factors With the Risk of Herpes Zoster: A Nationwide Population-Based Cohort Study
Source: Am J Epidemiol. 2021 Feb 11;190(6):1064–74. doi: 10.1093/aje/kwab027 (PMC8168175; doi:10.1093/aje/kwab027)
Supplement: Web_Material_kwab027 [file web_material_kwab027.pdf]

Web Material

Lifestyle and Anthropometric Factors and Risk of Herpes Zoster: A Nationwide Population-Based Cohort Study

Sigrun A. J. Schmidt, Henrik Toft Sørensen, Sinéad M. Langan, and Mogens Vestergaard

Table of Contents

- **Web Appendix.** Additional description of the Danish National Health Survey and databases used in the study.
- **Web Figure 1.** Directed acyclic graph for the study.
- **Web Figure 2.** Hazard ratios (95% confidence intervals) associated with study covariables based on routinely collected registry data among survey non-responders (n=68,044) and among persons in final study population (n=101,894).
- **Web Table 1.** Code lists for exposures, outcomes, and covariables.
- **Web Table 2.** Distribution of variables prior to excluding those with missing data.
- **Web Table 3.** Characteristics by missingness, overall and for individual exposures. Values are numbers (percentages).
- **Web Table 4.** Odds ratios (95% confidence intervals) for missingness associated with study variables, overall and for individual exposures.
- **Web Table 5.** Cohort characteristics at follow-up start, by smoking status.
- **Web Table 6.** Cohort characteristics at follow-up start, by alcohol consumption.
- **Web Table 7.** Cohort characteristics at follow-up start, by body mass index category.
- **Web Table 8.** Cohort characteristics at follow-up start, by physical activity.
- **Web Table 9.** Fully-adjusted hazard ratios (95% confidence intervals) for the association between lifestyle factors and herpes zoster, by age and sex.
- **Web Table 10.** Fully-adjusted hazard ratios (95% confidence intervals) for the association between body mass index category and herpes zoster, by physical activity.
- **Web Table 11.** Fully-adjusted hazard ratios (95% confidence intervals) for the association between physical activity and herpes zoster, by body mass index category.

- **Web Table 12.** Hazard ratios (95% confidence intervals) for the association between exposures and herpes zoster adjusting for covariables in the main model and additionally for working and civil status.
- **Web Table 13.** Hazard ratios (95% confidence interval) for the association between lifestyle and anthropometric factors and herpes Zoster, 2010 Danish National Health Survey, main analysis and sensitivity analysis adjusting additionally for the Charlson Comorbidity Index (CCI).

Web Appendix. Additional description of the Danish National Health Survey and databases used in the study

For the study, we linked data from the Danish National Health Survey to several Danish nationwide registries with routinely collected data concerning aspects of medical care and education. We linked data at the individual-level using the unique 10-digit central personal registration number, which is used when recording data across the public system. The data sources used are described below.

The Danish National Health Survey

The cohort included participants of the Danish National Health Survey (also called the “How Are You?” survey, or in Danish “Hvordan har du det”)—a nationwide cross-sectional survey conducted in 2010.[1] The Survey included six mutually exclusive random samples: five stratified samples from each of the five Danish Regions and one national sample.[1] The source population comprised all Danish inhabitants aged 16 years or older on January 1, 2010 (estimated at 4,460,874 persons [www.statistikbanken.dk]). Institutionalized individuals were also eligible. The Danish Civil Registration System, [2] which has assigned the unique central personal registration number to all Danish residents at birth or emigration since 1968, was used for sampling.

In total, 298,850 persons were invited by a mailed survey letter including a paper questionnaire.[1] A link to an online version was provided as an option to the paper questionnaire in all but one sample (Central Denmark Region). Data were collected in the period February to April 2010. Postal reminders were distributed at least twice. The overall response proportion was 60% (n=177,639), varying between 52% and 66% in the six subsamples. As CPR numbers for all invited persons were available from the Survey, subsequent linkage to nationwide Danish registries has enabled comparison of responders and non-responders. Thus, it has been shown that non-responders were more likely to be male, unmarried, and have non-Danish ethnic background.[1] Statistics Denmark, the central authority on Danish statistics, has computed calibrated weights for non-response using information on various characteristics defined through linked data.[1,3] Thus, each person in the survey has been assigned a calibrated weight based on the likelihood of response from a person with the same age, sex, municipality of residence, highest complete educational level, income, marital status, ethnic background, number of visits to the general practitioner in 2007, a hospitalization in 2007 (yes/no), occupational status, owner/tenant status, and protection from inquiries during statistical and scientific surveys for all individuals living in Denmark on 1 January 2010.[1,3] The sampling technique is accounted for in the weights by weighting the municipality of

residence according to its population size. We included the calibrated weights to statistically allow for study design and differential non-response.

A standard questionnaire with 52 core questions within the domains sociodemographic characteristics, health-related quality of life, health behavior, morbidity, consequences of illness, and social relations was used in all six samples comprising the Danish National Health Survey. Furthermore, the authority responsible for each sample also had the option to add questions of interest.

For the current study, we have access to a large number of variables, but not all, from the survey for the population aged 25 years or older on January 1, 2010. Through the servers of Statistics Denmark, we linked the survey data to records in the Civil Registration System,[2] the Danish National Patient Registry[5] the Danish Central Psychiatric Research Registry,[6] Danish National Prescription Registry,[7] and the Population Education Registry.[8] As the exact date for when the participant filled out the questionnaire is not available, we started follow-up on May 1, 2010 for all persons, excluding those with inactive status in the CRS on this date.

The Danish Civil Registration System

The Civil Registration System is key to Danish registry-based research because it is responsible for assigning the Central Personal Registration number and provides a complete account of the entire Danish population on a day-to-day basis.[2] It was established in 1968 and records dates of birth, death and emigration, citizenship, place of birth, and many other variables for all residents. The Civil Registration System was used for sampling for the questionnaire, to follow the study cohort and to define certain variables (age, sex, ethnicity) in the current study.

The Danish National Patient Registry and *the Danish Central Psychiatric Research Registry*

Data on hospital diagnoses and treatments in Denmark are recorded in two discharge registries, the Danish National Patient Registry[5] and the Danish Psychiatric Central Registry,[6] which were merged in 1995. These registries provide data on all psychiatric admissions since 1970, somatic admissions since 1978, and contacts to psychiatric and somatic hospital outpatient clinics and emergency rooms since 1995.[5,6] At the end of each contact, a primary diagnosis (the main reason for contact) and optional secondary (contributing) diagnoses are recorded by the physician in charge.[5] Diagnoses were coded using the International Classification of Diseases (ICD), 8th revision until 1994, and ICD-10 thereafter. Surgical and non-surgical procedures and treatments are also registered.

The Danish National Prescription Registry

The Danish National Prescription Registry includes anonymized data on prescriptions filled at Danish community pharmacies since January 1, 1995.[7] When a prescription is filled at the pharmacy, the patient's CPR number, dispensing details for the drug, and identifiers for the issuing physician and the pharmacy are logged by the electronic accounting system and transferred to the registry. Indication and dosing instructions are registered since April 2004, but are recorded inconsistently.

The Population Education Registry

The Population Education Registry registers attained education for residents.[8] It is based on administrative records from educational institutions and is supplemented with self-reported data for individuals completing education before 1974 and immigrants with schooling outside Denmark. In 2007, 3% of ethnic Danes born in 1945–1990 had missing data, and up to 15% of immigrants.

References

1 Christensen AI, Ekholm O, Glümer C, *et al.* The Danish National Health Survey 2010. Study design and respondent characteristics. *Scand J Public Health* 2012; **40**: 391–7.

2 Schmidt M, Pedersen L, Sørensen HT. The Danish Civil Registration System as a tool in epidemiology. *Eur J Epidemiol* 2014; **29**: 541–9.

3 Fangel S, Linde PC, Thorsted BL. Nye problemer med repraesentativitet i surveys, som opregning med registre kan reducere. Metode og Data, 2007.

4 Morgan ES, Umberson K, Hertzog C. Construct validation of self-reported stress scales. *Psychological Assessment* 2014; **26**: 90–9.

5 Schmidt M, Schmidt SAJ, Sandegaard JL, Ehrenstein V, Pedersen L, Sørensen HT. The Danish National Patient Registry: A review of content, data quality, and research potential. *Clin Epidemiol* 2015; **7**: 449–90.

6 Mors O, Perto GP, Mortensen PB. The Danish Psychiatric Central Research Register. *Scand J Public Health* 2011; **39**: 54–7.

7 Pottegård A, Schmidt SAJ, Wallach-Kildemoes H, Sørensen HT, Hallas J, Schmidt M. Data Resource Profile: The Danish National Prescription Registry. *Int J Epidemiol* 2017; **46**: 798–798f.

8 Jensen VM, Rasmussen AW. Danish Education Registers. *Scand J Public Health* 2011; **39**: 91–4.

9 Pedersen CB. The Danish Civil Registration System. *Scand J Public Health* 2011; **39**: 22–5.

Web Figure 1. Directed acyclic graph for the study.

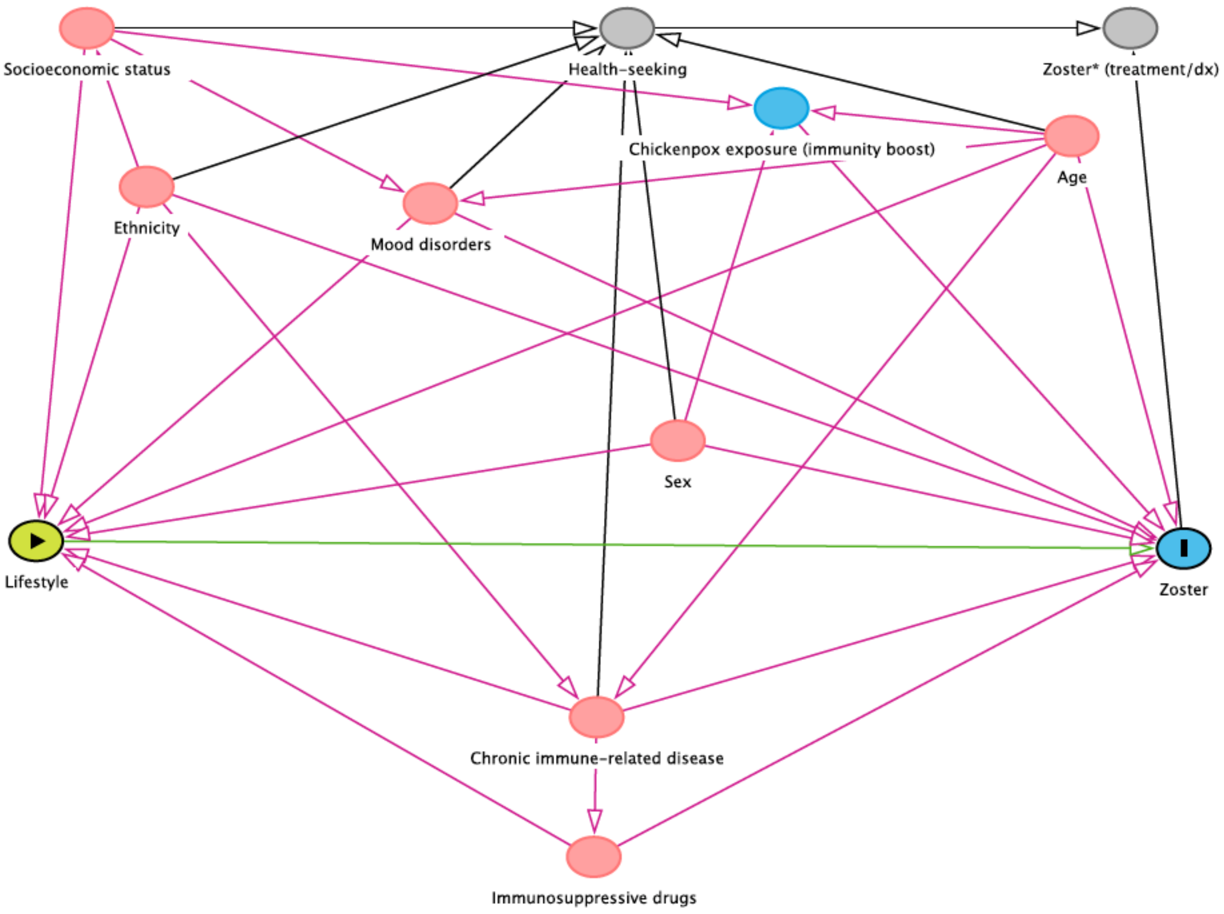

Note: This figure is a simplified graph where we have combined demographic, lifestyle and anthropometric factors, various chronic immune-mediated diseases, and immunosuppressive drugs in three variables. The graph was generated in the online program DAGitty v3.0 (available on dagitty.net). Exposure (lifestyle factors) is represented by the green circle ( ) and the outcome (zoster) by the blue circle with the I ( ). Blue circles ( ) represent variables that are ancestors of the outcome and red circles ( ) those that are ancestors of both exposure and outcome. Green arrows represent causal pathways and pink arrows represent biasing paths.

Based on the graph, a minimal sufficient adjustment set for estimating the total and direct effect includes age, sex, chronic immune-related disease, immunosuppressive drugs, mood disorder, ethnicity, and socioeconomic status.

Note that some associations could work in both directions. The figure shows that being diagnosed and/or treated for a chronic immune-related disease may affect health-related behavior and motivate people to change their lifestyle. However, it is also possible that lifestyle (*e.g.*, smoking) affects the risk of certain chronic immune-related diseases and the decision to treat with certain immunosuppressive drugs (*e.g.*, methotrexate should be avoided with alcohol). In that situation (an arrow from lifestyle to chronic immune-related disease and immunosuppressive drugs), the minimal sufficient adjustment set for the total effect includes age, sex, mood disorders, ethnicity, and socioeconomic status and for the direct effect it includes additionally chronic immune-related disease and immunosuppressive drugs. Note that for physical exercise the effect on zoster risk may be partly mediated through body mass index (or vice versa), which is not shown in the figure.

Web Figure 2. Hazard ratios (95% confidence intervals)[a] associated with study covariables based on routinely collected registry data among survey non-responders (n=68,044) and among persons in final study population (n=101,894).

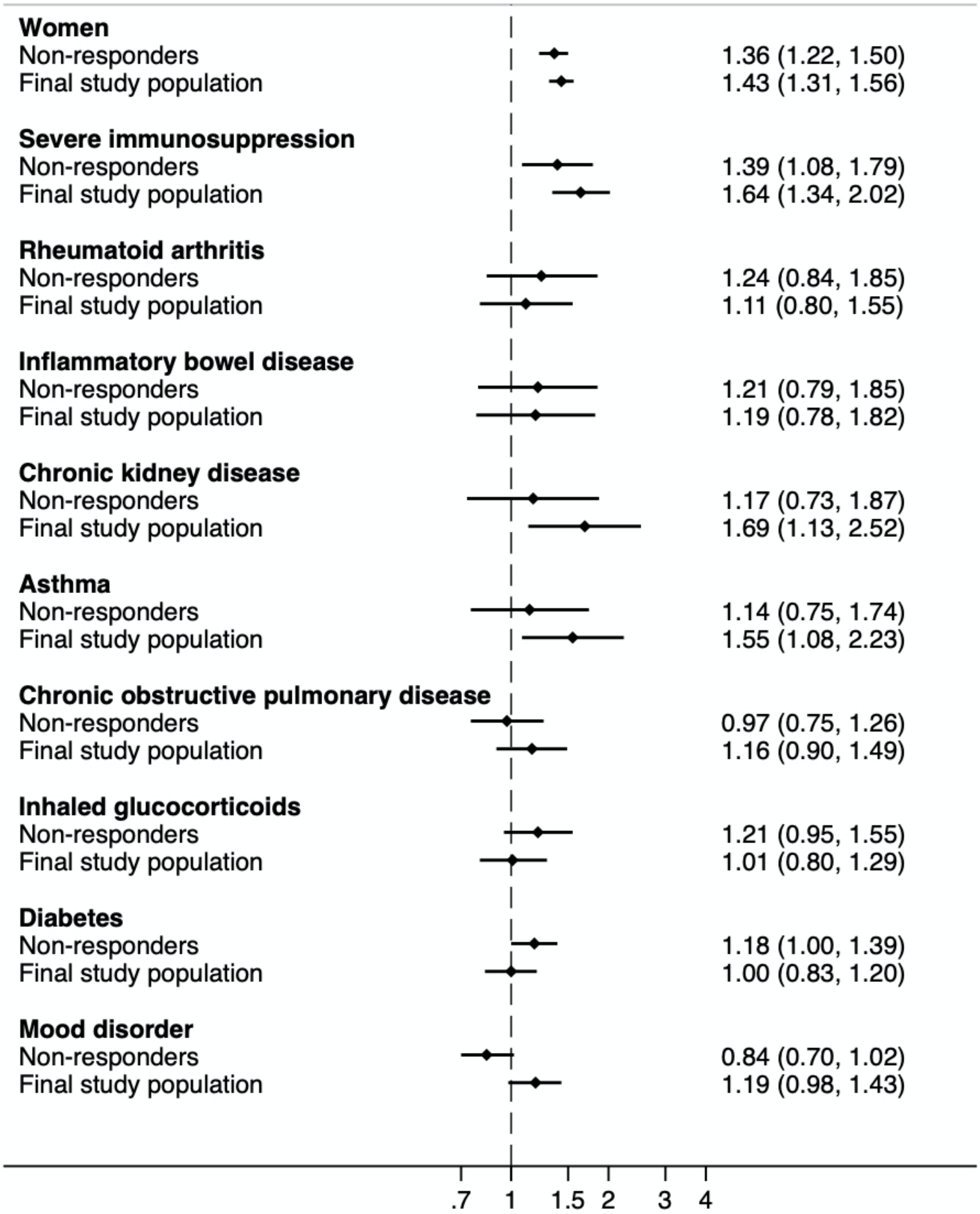

[a]Computed using Cox regression with age as underlying time scale and stratified by birth cohort. Each variable was mutually adjusted for other variables in the figure. Note: Includes non-responders fulfilling the same eligibility criteria as the final study population (*i.e.*, age 40 years or older at baseline, no previous diagnosis of zoster or postherpetic neuralgia; and no previous prescription for acyclovir, valacyclovir, or famciclovir at any dose). Survey information was not used for defining covariables, as this information was not available for non-responders.

Results for systemic lupus erythematosus not shown because of low number.

Web Table 1. Code lists for exposures, outcomes, and covariables.

Definitions of exposures

Variable	Definition	Source in the survey
Smoking status	We categorized persons as never smokers (reference group), former smokers, or current smokers (daily or non-daily).	Based on the question: "Do you smoke?" with the following options for response:<br>1: Yes, every day<br>2: Yes, at least once a week<br>3: Yes, but less than once a week<br>4: No, I quit<br>5: No, I have never smoked<br><br>Coded as:<br>Never smokers: if response 5<br>Former smoker: if response 4<br>Current smoker: if response 1-3
Average daily tobacco consumption	Among current daily smokers reporting level of tobacco consumption, we calculated the total daily consumption in grams using the following rule: 1 cigarette equals 1 gram, 1 cigar equals 4.5 gram, 1 cheroot equals 3 gram, and 1 pipe stop equals 3 gram of tobacco. We the grouped the total consumption by 5-gram increments: 1–5, 6–10, 11–15, 16–20, 21–25, 26–30, 30+ grams. Never smokers provided the reference group.	Based on four questions for "How many [cigarettes/cheroots/cigars/pipe loads] do you smoke on average per day". Only current daily smokers were asked these questions.
Weekly alcohol consumption	Based on number of standardized alcohol units consumed weekly, we categorized drinking as low-risk (≤7 for women/≤14 for men); intermediate-risk (8–14 for women/15–21 for men); or high-risk (≥15 for women/≥22 for men). We included non-drinkers in the low-risk group, which was the reference group.	Based on questions about the number of units each day during one week. The question was "How many units do you typically drink on each of the days during the week? Start with Monday and take one day at a time (fill in all fields, even if the answer is 0)". The participant was then asked to specify the number of units of beer/alcohol cider, wine/fortified wines and spirits/alcopops, respectively, for each of the seven days a week. We computed the total number of units per week, rounded and then categorized into the three categories while accounting for sex.<br><br>Prior to the question about level of consumption, participants were asked "Have you consumed alcohol within the past year?". Those that responded "No" were not asked to fill in information about level of consumption.
Binge drinking	We defined binge drinkers as those who reported daily, almost daily, or weekly intake of ≥5 units of alcohol on a single occasion. Those reporting binge drinking monthly or less, including non-drinkers, provided the reference group.	Based on the question "How often do you drink 5 or more units of alcohol on a single occasion?" with the following response options:<br>1: Almost daily or daily<br>2: Weekly<br>3: Monthly<br>4: Rarely<br>5: Never<br>Persons who responded that they had not consumed alcohol within the past year were not asked to respond to this question.

BMI category	We categorized BMI according to the WHO definition: underweight (BMI<18.5 kg/m^2); normal weight (18.5≤BMI<25 kg/m^2; referent); overweight (25≤BMI<30 kg/m^2); or obese (BMI≥30 kg/m^2). In a restricted cubic splines model, we included BMI as a continuous variable, rounding to the nearest full integer, capped at 60+ kg/m^2, with BMI = 22 as the referent.	BMI was computed as weight (kg) divided by height (m)^2 based on the response to the following two questions:<br>"How tall are you (without shoes)?" (in cm)<br>"How much do you weigh the whole kilos (without clothes)?" (in kilos)
Physical activity	We categorized physical activity as sedentary; light (referent); or moderate/vigorous.	Based on the question: "Looking back at the past year, how would you best describe your physical activity during leisure time?" with the following response options:<br>1: Perform heavy exercise and competitive sports regularly and several times a week<br>2: Perform recreational sports or heavy gardening or similar activity at least 4 hours per week<br>3: Walking, cycling or other light exercise at least 4 hours a week (includes Sunday excursions, light gardening and cycling or walking to work)<br>4: Reading, watching television or other sedentary activity<br><br>We then categorized physical activity as:<br>Sedentary: if response 4<br>Light: if response 3<br>Moderate/vigorous: if response 2 or 1

Definitions of outcome (herpes zoster)

Type of record	Codes
Hospital diagnoses of herpes zoster	ICD-8: 053;<br>ICD-10: DB02, DG051I, DG051M, DH031F, DH131M, DH192D, DH192J, DH220C, DH621B
Antiviral treatment in general practice	
Acyclovir	ATC code: J05AB01; Zoster specific doses (800 mg in packages of 35 pills) identified by Nordic article numbers 005404, 007109, 044597, 057554, 078015, 082158, 106864, 149871, 397653, 434183, 445715, 447144, 470021, 480533, 496455, 515258, 516328, 560359, and 587160,
Valacyclovir	ATC code: J05AB011; Zoster specific doses (500 mg tablet doses) identified by **excluding** prescriptions with Nordic article number 025929, 030449, 172940, 447695, 498063, 534343, or 540242
Famciclovir	ATC code: J05AB09; Zoster specific doses (500 mg tablet doses) identified by Nordic article numbers 088196, 455584, 494756, 548975 and 550906
Hospital diagnoses of postherpetic neuralgia	ICD-10: DG530

Abbreviations: ICD=International Classification of Disease; ATC=Anatomical Therapeutic Chemical Classification System

Definitions of covariables

Variable	Definition	Codes
**Immunosuppressive and chronic diseases associated with herpes zoster**		
Rheumatoid arthritis	– Any previous diagnosis in the DNPR *or*<br>– Self-reported in the questionnaire	– ICD-8: 712.09, 712.19, 712.29, 712.39, 712.59<br>– ICD-10: DG737D, DI328A, DI398E, DI418A, DI528A, DJ990, DM05, DM060, DM061, DM062, DM063, DM068, DM069, DM080, DM082, DM083, DM084<br>– Questionnaire: Included those with response 2 or 3 to the question: "Do you or have you had rheumatoid arthritis?" where options were<br>1: No, I have never had it<br>2: Yes, I have it now<br>3: Yes, I have previously had it<br>– Questionnaire: Included those with response 1 to the question: "If you have had rheumatoid arthritis, do you have sequela?" where options were :<br>1: Yes<br>2: No
Systemic/subacute lupus erythematosus	– Any previous diagnosis in the DNPR	– ICD-8: 734.19;<br>– ICD-10: DL931, DG058A, DG737C, DI328B, DI398C, DJ991C, DL932, DM32, DN085A, DN164B
Inflammatory bowel disease	– Any previous diagnosis in the DNPR	– ICD-8: 563.01, 563.19, 569.04;<br>– ICD-10: DK50, DK51, DM074, DM075, DM091, DM092
Chronic kidney disease	– Any previous record of chronic kidney disease stage 3 or higher, renal failure, chronic uremia, dialysis or renal transplantation in the DNPR	– ICD-8: 584, 792, 997.7, Y95.09<br>– ICD-10: DL298C, DG638A, DE853B, DT825A, DT825B, DT825C, DT856C, DI120, DI131, DI132, DI770, DN165, DN180, DN183, DN184, DN185, DN188, DN189, DN19, DT824, DT861, DZ49, DZ94, DZ992, DT817E1<br>– Surgery codes: KJAK10, KJAK11, KJAK13, KJAK14, KTJA30, KTJA32, KTJA35, KKAS<br>– Treatment codes: BJFD2, BJFZ, BJKB, BUFC1, BWDC5, ZZ0151A, ZZ4341, ZZ4342, ZZ4343, ZZ4346, ZZ4347, ZZ4348, ZZ4350
Active asthma	– Any previous diagnosis in the DNPR or self-reported diagnosis in the questionnaire *and*<br>– Asthma prescription in the Prescription Registry in the year before follow-up start *and*<br>– No previous COPD defined as in previous variable	– ICD-8: 493<br>– ICD-10: DJ45, DJ46<br>– Questionnaire: Included those with response 2 or 3 to the question: "Do you or have you had asthma?" where options were<br>1: No, I have never had it<br>2: Yes, I have it now<br>3: Yes, I have previously had it<br>– Questionnaire: Included those with response 1 to the question: "If you have had asthma, do you have sequela?" where options were :<br>1: Yes<br>2: No<br>ATC: R03
Chronic obstructive pulmonary disease	– Any previous diagnosis in the DNPR *and* ≥35 years at first diagnosis (all identified through questionnaire will be ≥40 years) *or*<br>– Self-reported diagnosis in the questionnaire	– ICD-8: 491, 492<br>– ICD-10: DJ41, DJ42, DJ43, DJ44<br>– Questionnaire: Included those with response 2 or 3 to the question: "Do you or have you had chronic bronchitis, emphysema, or COPD?" where options were<br>1: No, I have never had it<br>2: Yes, I have it now<br>3: Yes, I have previously had it<br>– Questionnaire: Included those with response 1 to the question: "If you have had chronic bronchitis, emphysema, or COPD, do you have sequela?" where options were :<br>1: Yes

		– 2: No
Inhaled glucocorticoids	– Any record in the Prescription Registry within 90 days before start of follow-up	– ATC: R03BA, R03AK06, R03AK07, R03AK08, R03AK09, R03AK10, R03AK11
Diabetes mellitus	– Any previous diagnosis in DNPR *or*<br>– Self-reported diabetes in the questionnaire, *or*<br>– ≥2 prescriptions for antidiabetics (except women treated with metformin alone at age 20 to 39 years, as that may represent treatment of polycystic ovarian syndrome)	– ICD-8: 249, 250<br>– ICD-10: DE10, DO240, DE11, DO241<br>– Questionnaire: Included those with response 2 or 3 to the question: “Do you or have you had diabetes?” where options were<br>1: No, I have never had it<br>2: Yes, I have it now<br>3: Yes, I have previously had it<br>– Questionnaire: Included those with response 1 to the question: “If you have had diabetes, do you have sequela?” where options were:<br>1: Yes<br>2: No<br>– ATC: A10A, A10B, B04AX07, C10AX04 (excluding A10BE01**)**
Mood disorder (moderate/severe depression, anxiety, or stress and adjustment disorder)	– Any previous diagnosis in the DNPR or the Central Psychiatric Registry before start of follow-up, including also unspecific depression diagnoses such as depression in dementia.	– ICD-8: 296.09, 296.29, 296.99, 298.09, 300.49, 300.09, 300.19, 300.29, 295.79<br>– ICD-10: DF00x3, DF01x3, DF02x3, DF0393, DF0632, DF064, DF1x54, DF204, DF25, DF32, DF33, DF341, DF40, DF41, DF43, DF3810, DF530, DO993B2, DO993B3
Severe immunosuppression		
Human immunodeficiency virus infection	– Any previous diagnosis in the DNPR	– ICD-8: 079.83<br>– ICD-10: DB20, DB21, DB22, DB23, DB24, DZ21
Hematopoietic stem cell or bone marrow transplantation	– Any previous diagnosis in the DNPR	– ICD-8: None<br>– ICD-10: DT860, DZ948C, DZ948 (if not DZ948A, DZ948B, DZ948C and if coded as a B-diagnosis or additional diagnosis together with one of the following primary diagnoses: DC770, DC81–DC96, DD45–DD47, DD50–DD85, DD87–DD89, DT860, DT860A, or DT888N)<br>– Treatment codes: BOQE, BOQF
Other cellular immune deficiency	– Any previous diagnosis in the DNPR	– ICD-8: 284.01, 284.02, 284.08, 284.09, 758.30<br>– ICD-10: DD611, DD612, DD613, DD618, DD619, DD81, DD820, DD821, DD822, DD83, DD830, DD831, DD832, DD838, DD839
Leukemia	– Any previous diagnosis in the DNPR within two years before start of follow-up	– ICD-8: 204, 205, 206, 207<br>– ICD-10: DC91, DC92, DC93, DC94, DC95
Lymphoma	– Any previous diagnosis in the DNPR within two years before start of follow-up	– ICD-8: 200, 201, 202<br>– ICD-10: DC81, DC82, DC83, DC84, DC85, DC86, DC88, DC96
Myeloma	– Any previous diagnosis in the DNPR within two years before start of follow-up	– ICD-8: 203<br>– ICD-10: DC90
Oral glucocorticoids	– Any record in the Prescription Registry	– H02AB (excluding Nordic article numbers for injections)

	within 90 days before start of follow-up	
Other immunosuppressant drugs	– A prescription in the Prescription Registry or treatment code in the DNPR within 90 days before start of follow-up	– ATC: ML01, ML04, MV02CA01, or MV02CA02 used as additional code in the DNPR<br>– Treatment codes: BOHJ, BWHA, BWHB<br>– ATC: L01, L04, V02CA01, V02CA02 in Prescription Registry
Charlson Comorbidity Index	– Based on first-time records in DNPR and the Prescription Registry within 1 year before baseline	– See table below for coding of disease categories and their corresponding scores
**Socio-demographic variables**		
Age	– Age at baseline based on birth date from the Civil Registration System	
Sex	– From the Civil Registration System	
Ethnicity	– Categorized as Danish, other Western, or non-Western based on information on country of origin.	Country of origin is based on citizenship, place of birth, and parents' place of birth, as recorded in the Civil Registration System. The following algorithm is used:<br>– When neither parents is known, the country of origin is defined based on personal information only. Thus, for immigrants, the country of birth is used. If persons are descendants, it is assumed that the country of origin is equal to the country of citizenship.<br>– When only one parent is known, the country of origin is defined by that parent's country of birth. If this is Denmark, the citizenship country is used.<br>– When both parents are known, the country of origin is defined based on the mother's country of birth or country of citizenship, respectively.<br>Western countries include the countries of the European Union, Andorra, Australia, Canada, Iceland, Liechtenstein, Monaco, New Zealand, Norway, San Marino, Switzerland, the United States and the Vatican City. Non-Western countries include the European countries Albania, Bosnia and Herzegovina, Belarus, Yugoslavia, Kosovo, Macedonia, Moldova, Montenegro, Russia, Serbia, the Soviet Union, Turkey and Ukraine; all countries in Africa, South and Central America and Asia; all countries in Oceania (near Australia and New Zealand); and stateless people.
Education	– Short (<10 years), intermediate (10–15 years), high education (>15 years), according to the United Nations Educational, Scientific and Cultural Organization's (UNESCO's) classification[1]	Based on the variable 'hfaudd recorded at Statistics Denmark. The 'hfaudd' specifies the highest achieved education, determined using the main categories of the Danish nomenclature for education, DISCED. By conversion to a specific format ('afsp4e'), we determined length of education categorized as:<br>– Short education (basic school and special needs education), corresponding to less than 10 years of education.<br>– Intermediate education (upper secondary education, vocationally oriented education and training etc., bachelor programs; professional bachelor programs; academy profession programs), corresponding to >10 to 15 years of education.<br>– High education (Master's programs and PhD), corresponding to >15 years of education
Working status	– "Working" or "Not working" based on self-reported current working status in the questionnaire	Working: if response "employed" in questionnaire<br>Not working: if response in questionnaire was "unemployed", "disability pensioner", "early or public retirement pensioner", or "other/student"

Civil status	– Cohabitating or single based on information recorded in the Civil Registration System	Defined according to an algorithm generated by Statistics Denmark, which identifies a person as being single or in partnership (married or cohabitating) based on information on civil status, demographics, exact address, and kinship (*e.g.*, personal identifiers of children).[2]
**Other variables**		
Calibrated survey weights	– Weight enumerating the sample size, proportional to weight_pop (variable weighing the population)	See description of the calibrated weights in Web Appendix section.

Abbreviations: DNPR=Danish National Patient Registry; ICD=International Classification of Disease; ATC=Anatomical Therapeutic Chemical Classification System

For surgery and treatment codes, we included both main codes and additional codes (i.e., C_OPR/C_TILOPR).

[1]United Nations Educational, Scientific and Cultural Organization. International Standard Classification of Education (ISCED). 2011. Available at: http://uis.unesco.org/sites/default/files/documents/international-standard-classification-of-education-isced-2011-en.pdf. Accessed April 14, 2020.

[2]Documentation available in Danish at:
https://www.dst.dk/da/Statistik/dokumentation/Times/forskningsservice/efalle
https://www.dst.dk/da/Statistik/dokumentation/Times/cpr-oplysninger/c-faelle-id

Coding of the Charlson Comorbidity Index

Score 1	
Myocardial infarction	ICD-8: 410; ICD-10: I21, I22, I23
Congestive heart failure	ICD-8: 427.09, 427.10, 427.11, 427.19, 428.99, 782.49; ICD-10: I50, I11.0, I13.0, I13.2
Peripheral vascular disease	ICD-8: 440, 441, 442, 443, 444, 445; ICD-10: I70, I71, I72, I73, I74, I77
Cerebrovascular disease	ICD-8: 430-438; ICD-10: I60-I69, G45, G46
Dementia	ICD-8: 290.09-290.19, 293.09; ICD-10: F00-F03, F05.1, G30
Chronic pulmonary disease	ATC: two redemptions with 6 weeks or less apart for any of the respiratory medications R03A, R03B, R03C, R03D; ICD-8: 490-493, 515-518, ICD-10: J40-J47, J60-J67, J68.4, J70.1, J70.3, J84.1, J92.0, J96.1, J98.2, J98.3
Connective tissue disease	ICD-8: 712, 716, 734, 446, 135.99; ICD-10: M05, M06, M08, M09, M30, M31, M32, M33, M34, M35, M36, D86
Ulcer disease	ICD-8: 530.91, 530.98, 531-534; ICD-10: K22.1, K25-K28
Mild liver disease	ICD-8: 571, 573.01, 573.04; ICD-10: B18, K70.0-K70.3, K70.9, K71, K73, K74, K76.0
Diabetes types 1 and 2	Defined as above except self-reported diabetes in the questionnaire was not included as date of diagnosis was not available for these.
Score 2	
Hemiplegia	ICD-8: 344; ICD-10: G81, G82
Moderate to severe renal disease	ICD-8: 403, 404, 580-583, 584, 590.09, 593.19, 753.10-753.19, 792; ICD-10: I12, I13, N00-N05, N07, N11, N14, N17-N19, Q61
Diabetes with end-organ damage	ICD-8: 249.01-249.05, 249.08, 250.01-250.05, 250.08; ICD-10: E10.2-E10.8, E11.2-E11.8, E14.2, E14.8
Any tumor except skin cancer	ICD-8: 140-194; ICD-10: C00-C75
Leukemia	ICD-8: 204-207; ICD-10: C91-C95
Lymphoma	ICD-8: 200-203, 275.59; ICD-10: C81-C85, C88, C90, C96
Score 3	
Moderate to severe liver disease	ICD-8: 070.00, 070.02, 070.04, 070.06, 070.08, 573.00, 456.00-456.09; ICD-10: B15.0, B16.0, B16.2, B19.0, K70.4, K72, K76.6, I85
Score 6	
Metastatic solid tumor	ICD-8: 195-198, 199; ICD-10: C76-C80
AIDS	ICD-8: 079.83; ICD-10: B21-B24

ICD=International Classification of Disease; ATC=Anatomical Therapeutic Chemical Classification System

Web Table 2. Distribution of variables prior to excluding those with missing data.

	n	%
**Total**	116,871	100
**Age group (years)**		
40–49	29,858	25.5
50–59	30,150	25.8
60–69	32,139	27.5
70–79	17,305	14.8
80+	7,419	6.3
**Sex**		
Men	55,949	47.9
Women	60,922	52.1
**Immunosuppressive and chronic diseases**		
Severe immunosuppression[a]	3,438	2.9
Rheumatoid arthritis	8,542	7.3
Systemic lupus erythematosus	116	0.1
Inflammatory bowel disease	1,382	1.2
Chronic kidney disease	796	0.7
Asthma	3,250	2.8
Chronic obstructive pulmonary disease	7,942	6.8
Inhaled corticosteroids	4,973	4.3
Diabetes	8,674	7.4
Mood disorder	5,691	4.9
**Highest achieved education[b]**		
Short	32,872	28.1
Intermediate	55,560	47.5
High	26,043	22.3
Missing	2,396	2.1
**Ethnicity**		
Danish	111,160	95.1
Other Western	2,959	2.5
Non-Western	2,752	2.4
**Smoking status**		
Never	46,205	39.5
Former	40,579	34.7
Current	26,856	23
Missing	3,231	2.8
**Alcohol consumption[c]**		
Low-risk	84,088	71.9
Intermediate-risk	15,575	13.3
High-risk	11,245	9.6
Missing	5,963	5.1
**Binge drinking weekly or more**		
No	10,3782	88.8
Yes	9,532	8.2
Missing	3,557	3.0
**Body mass index**		
Underweight	1,753	1.5
Normal	50,140	42.9
Overweight	43,481	37.2
Obese	17,718	15.2
Missing	3,779	3.2
**Physical activity**		
Sedentary	17,447	14.9
Light	70,824	60.6
Moderate	22,896	19.6
Vigorous	1,787	1.5
Missing	3,917	3.4

[a]Variables for severe immunosuppression were combined because of low numbers.
[b]Categorized as short (<10 years), intermediate (10–15 years), and high education (>15 years), according to the United Nations Educational, Scientific and Cultural Organization's (UNESCO's) classification
[c]Categorized based on number of standardized alcohol units consumed weekly: low-risk (≤7 for women/≤14 for men) intermediate-risk (8–14 for women/15–21 for men); or high-risk (≥15 for women/≥22 for men).

Web Table 3. Characteristics by missingness, overall and for individual exposures. Values are numbers (percentages).

	All variables		Smoking status		Alcohol consumption		Binge drinking		BMI category		Physical activity		Education	
	Non-missing	Missing	Non-missing	Missing	Non-missing	Missing	Non-missing	Missing	Non-missing	Missing	Non-missing	Missing	Non-missing	Missing
**Herpes zoster**	2635 (2.6)	450 (3.0)	2993 (2.6)	92 (2.8)	2896 (2.6)	189 (3.2)	2975 (2.6)	110 (3.1)	2974 (2.6)	111 (2.9)	2976 (2.6)	109 (2.8)	3012 (2.6)	73 (3.0)
**Age group (years)**														
40-49	27879 (27.4)	1979 (13.2)	29439 (25.9)	419 (13.0)	29234 (26.4)	624 (10.5)	29515 (26.0)	343 (9.6)	29329 (25.9)	529 (14.0)	29465 (26.1)	393 (10.0)	29554 (25.8)	304 (12.7)
50-59	27730 (27.2)	2420 (16.2)	29644 (26.1)	506 (15.7)	29336 (26.5)	814 (13.7)	29670 (26.2)	480 (13.5)	29535 (26.1)	615 (16.3)	29644 (26.2)	506 (12.9)	29783 (26.0)	367 (15.3)
60-69	28402 (27.9)	3737 (25.0)	31352 (27.6)	787 (24.4)	30645 (27.6)	1494 (25.1)	31285 (27.6)	854 (24.0)	31317 (27.7)	822 (21.8)	31160 (27.6)	979 (25.0)	31695 (27.7)	444 (18.5)
70-79	13611 (13.4)	3694 (24.7)	16506 (14.5)	799 (24.7)	15616 (14.1)	1689 (28.3)	16278 (14.4)	1027 (28.9)	16393 (14.5)	912 (24.1)	16192 (14.3)	1113 (28.4)	17023 (14.9)	282 (11.8)
80+	4272 (4.2)	3147 (21.0)	6699 (5.9)	720 (22.3)	6077 (5.5)	1342 (22.5)	6566 (5.8)	853 (24.0)	6518 (5.8)	901 (23.8)	6493 (5.7)	926 (23.6)	6420 (5.6)	999 (41.7)
**Sex**														
Men	49550 (48.6)	6399 (42.7)	54652 (48.1)	1297 (40.1)	53608 (48.3)	2341 (39.3)	54568 (48.2)	1381 (38.8)	54475 (48.2)	1474 (39.0)	54218 (48.0)	1731 (44.2)	54831 (47.9)	1118 (46.7)
Women	52344 (51.4)	8578 (57.3)	58988 (51.9)	1934 (59.9)	57300 (51.7)	3622 (60.7)	58746 (51.8)	2176 (61.2)	58617 (51.8)	2305 (61.0)	58736 (52.0)	2186 (55.8)	59644 (52.1)	1278 (53.3)
**Immunosuppressive and chronic diseases**														
Severe immunosuppression[a]	2826 (2.8)	612 (4.1)	3300 (2.9)	138 (4.3)	3154 (2.8)	284 (4.8)	3251 (2.9)	187 (5.3)	3275 (2.9)	163 (4.3)	3270 (2.9)	168 (4.3)	3352 (2.9)	86 (3.6)
Rheumatoid arthritis	7068 (6.9)	1474 (9.8)	8291 (7.3)	251 (7.8)	7996 (7.2)	546 (9.2)	8223 (7.3)	319 (9.0)	8187 (7.2)	355 (9.4)	8192 (7.3)	350 (8.9)	8244 (7.2)	298 (12.4)
Systemic lupus erythematosus	100 (0.1)	16 (0.1)	d	d	d	d	d	d	d	d	d	d	d	d
Inflammatory bowel disease	1209 (1.2)	173 (1.2)	1347 (1.2)	35 (1.1)	1314 (1.2)	68 (1.1)	1349 (1.2)	33 (0.9)	1346 (1.2)	36 (1.0)	1331 (1.2)	51 (1.3)	1356 (1.2)	26 (1.1)
Chronic kidney disease	607 (0.6)	189 (1.3)	756 (0.7)	40 (1.2)	716 (0.6)	80 (1.3)	737 (0.7)	59 (1.7)	743 (0.7)	53 (1.4)	734 (0.6)	62 (1.6)	760 (0.7)	36 (1.5)
Asthma	2874 (2.8)	376 (2.5)	3171 (2.8)	79 (2.4)	3103 (2.8)	147 (2.5)	3152 (2.8)	98 (2.8)	3163 (2.8)	87 (2.3)	3162 (2.8)	88 (2.2)	3201 (2.8)	49 (2.0)
Chronic obstructive pulmonary disease	6480 (6.4)	1462 (9.8)	7694 (6.8)	248 (7.7)	7324 (6.6)	618 (10.4)	7566 (6.7)	376 (10.6)	7594 (6.7)	348 (9.2)	7528 (6.7)	414 (10.6)	7706 (6.7)	236 (9.8)
Inhaled corticosteroids	4096 (4.0)	877 (5.9)	4791 (4.2)	182 (5.6)	4572 (4.1)	401 (6.7)	4701 (4.1)	272 (7.6)	4767 (4.2)	206 (5.5)	4718 (4.2)	255 (6.5)	4866 (4.3)	107 (4.5)
Diabetes	6910 (6.8)	1764 (11.8)	8314 (7.3)	360 (11.1)	7954 (7.2)	720 (12.1)	8235 (7.3)	439 (12.3)	8215 (7.3)	459 (12.1)	8176 (7.2)	498 (12.7)	8402 (7.3)	272 (11.4)

Mood disorder	4698 (4.6)	993 (6.6)	5498 (4.8)	193 (6.0)	5246 (4.7)	445 (7.5)	5422 (4.8)	269 (7.6)	5419 (4.8)	272 (7.2)	5432 (4.8)	259 (6.6)	5540 (4.8)	151 (6.3)
**Highest achieved education[b]**														
Short	26603 (26.1)	6269 (49.8)	31355 (28.1)	1517 (49.7)	29741 (27.3)	3131 (55.0)	30974 (27.9)	1898 (56.2)	31112 (28.0)	1760 (49.7)	30840 (27.8)	2032 (54.9)	N/A	N/A
Intermediate	50787 (49.8)	4773 (37.9)	54433 (48.9)	1127 (36.9)	53578 (49.3)	1982 (34.8)	54401 (49.0)	1159 (34.3)	54224 (48.9)	1336 (37.8)	54285 (49.0)	1275 (34.4)	N/A	N/A
High	24504 (24.0)	1539 (12.2)	25636 (23.0)	407 (13.3)	25462 (23.4)	581 (10.2)	25724 (23.2)	319 (9.4)	25600 (23.1)	443 (12.5)	25647 (23.2)	396 (10.7)	N/A	N/A
**Ethnicity**														
Danish	97515 (95.7)	13645 (91.1)	108143 (95.2)	3017 (93.4)	105533 (95.2)	5627 (94.4)	107831 (95.2)	3329 (93.6)	107577 (95.1)	3583 (94.8)	107493 (95.2)	3667 (93.6)	109414 (95.6)	1746 (72.9)
Other Western	2392 (2.3)	567 (3.8)	2892 (2.5)	67 (2.1)	2830 (2.6)	129 (2.2)	2890 (2.6)	69 (1.9)	2872 (2.5)	87 (2.3)	2887 (2.6)	72 (1.8)	2643 (2.3)	316 (13.2)
Non-Western	1987 (2.0)	765 (5.1)	2605 (2.3)	147 (4.5)	2545 (2.3)	207 (3.5)	2593 (2.3)	159 (4.5)	2643 (2.3)	109 (2.9)	2574 (2.3)	178 (4.5)	2418 (2.1)	334 (13.9)
**Smoking status**														
Never	41608 (40.8)	4597 (39.1)	N/A	N/A	44297 (40.7)	1908 (38.8)	45129 (40.7)	1076 (40.1)	44883 (40.6)	1322 (42.6)	44998 (40.7)	1207 (38.4)	45316 (40.7)	889 (40.1)
Former	36339 (35.7)	4240 (36.1)	N/A	N/A	38745 (35.6)	1834 (37.3)	39583 (35.7)	996 (37.1)	39510 (35.7)	1069 (34.4)	39493 (35.7)	1086 (34.6)	39771 (35.7)	808 (36.5)
Current	23947 (23.5)	2909 (24.8)	N/A	N/A	25678 (23.6)	1178 (23.9)	26244 (23.7)	612 (22.8)	26143 (23.7)	713 (23.0)	26009 (23.5)	847 (27.0)	26337 (23.6)	519 (23.4)
**Alcohol consumptionc**														
Low-risk	76812 (75.4)	7276 (80.7)	82293 (75.7)	1795 (82.0)	N/A	N/A	83883 (75.8)	205 (74.0)	81840 (75.7)	2248 (81.0)	81820 (75.7)	2268 (81.7)	82348 (75.7)	1740 (81.8)
Intermediate-risk	14623 (14.4)	952 (10.6)	15329 (14.1)	246 (11.2)	N/A	N/A	15550 (14.1)	25 (9.0)	15275 (14.1)	300 (10.8)	15311 (14.2)	264 (9.5)	15380 (14.1)	195 (9.2)
High-risk	10459 (10.3)	786 (8.7)	11098 (10.2)	147 (6.7)	N/A	N/A	11198 (10.1)	47 (17.0)	11017 (10.2)	228 (8.2)	11002 (10.2)	243 (8.8)	11053 (10.2)	192 (9.0)
**Binge drinking weekly or more**														
No	93128 (91.4)	10654 (93.3)	101576 (91.5)	2206 (93.6)	101265 (91.5)	2517 (93.8)	N/A	N/A	100995 (91.5)	2787 (93.5)	100998 (91.5)	2784 (93.5)	101709 (91.5)	2073 (93.6)
Yes	8766 (8.6)	766 (6.7)	9380 (8.5)	152 (6.4)	9366 (8.5)	166 (6.2)	N/A	N/A	9337 (8.5)	195 (6.5)	9340 (8.5)	192 (6.5)	9390 (8.5)	142 (6.4)
**Body mass index**														
Underweight	1467 (1.4)	286 (2.6)	1707 (1.5)	46 (1.8)	1620 (1.5)	133 (2.7)	1680 (1.5)	73 (2.6)	N/A	N/A	1676 (1.5)	77 (2.7)	1669 (1.5)	84 (3.9)
Normal	45376 (44.5)	4764 (42.5)	49059 (44.4)	1081 (42.3)	48107 (44.5)	2033 (41.0)	48953 (44.4)	1187 (43.0)	N/A	N/A	48932 (44.4)	1208 (42.1)	49165 (44.3)	975 (45.2)
Overweight	39255 (38.5)	4226 (37.7)	42528 (38.5)	953 (37.3)	41576 (38.4)	1905 (38.4)	42445 (38.5)	1036 (37.5)	N/A	N/A	42371 (38.4)	1110 (38.6)	42723 (38.5)	758 (35.2)

Obese	15796 (15.5)	1922 (17.2)	17242 (15.6)	476 (18.6)	16829 (15.6)	889 (17.9)	17254 (15.6)	464 (16.8)	N/A	N/A	17241 (15.6)	477 (16.6)	17379 (15.7)	339 (15.7)
**Physical activity**														
Sedentary	14366 (14.1)	3081 (27.9)	16877 (15.3)	570 (23.2)	16179 (15.0)	1268 (26.3)	16744 (15.2)	703 (26.9)	16615 (15.1)	832 (30.4)	N/A	N/A	16523 (14.9)	924 (42.3)
Light	64493 (63.3)	6331 (57.2)	69381 (62.8)	1443 (58.8)	67962 (62.9)	2862 (59.4)	69284 (62.8)	1540 (58.9)	69304 (62.9)	1520 (55.6)	N/A	N/A	69820 (63.0)	1004 (46.0)
Moderate	21400 (21.0)	1496 (13.5)	22498 (20.4)	398 (16.2)	22286 (20.6)	610 (12.7)	22565 (20.5)	331 (12.7)	22547 (20.5)	349 (12.8)	N/A	N/A	22664 (20.5)	232 (10.6)
Vigorous	1635 (1.6)	152 (1.4)	1744 (1.6)	43 (1.8)	1706 (1.6)	81 (1.7)	1745 (1.6)	42 (1.6)	1754 (1.6)	33 (1.2)	N/A	N/A	1765 (1.6)	22 (1.0)

Abbreviations: N/A, not applicable

[a]Variables for severe immunosuppression were combined because of low numbers. The higher prevalence among those with missing data was driven by difference for oral glucocorticoids.

[b]Categorized as short (<10 years), intermediate (10–15 years), and high education (>15 years), according to the United Nations Educational, Scientific and Cultural Organization's (UNESCO's) classification

[c]Categorized based on number of standardized alcohol units consumed weekly: low-risk (≤7 for women/≤14 for men) intermediate-risk (8–14 for women/15–21 for men); or high-risk (≥15 for women/≥22 for men).

[d]Not shown because of low numbers.

Web Table 4. Odds ratios (95% confidence intervals) for missingness associated with study variables, overall and for individual exposures.

	**Overall[a]**	**Smoking status**	**Alcohol consumption**	**Binge drinking**	**BMI category**	**Physical activity**	**Education**
**Observations**	116,871	103,892	105,987	105,987	106,690	106,690	103812
**Herpes zoster**	0.92 (0.82–1.02)	0.80 (0.59–1.09)	1.03 (0.86–1.24)	0.95 (0.73–1.22)	0.83 (0.64–1.07)	0.98 (0.78–1.24)	0.97 (0.73–1.28)
**Age group (years)**							
40–49	1 (ref.)	1 (ref.)	1 (ref.)	1 (ref.)	1 (ref.)	1 (ref.)	1 (ref.)
50–59	1.26 (1.18–1.34)	1.21 (1.04–1.41)	1.20 (1.06–1.36)	1.36 (1.15–1.61)	1.05 (0.92–1.19)	1.28 (1.10–1.50)	1.33 (1.12–1.57)
60–69	1.96 (1.85–2.08)	1.63 (1.41–1.89)	1.99 (1.78–2.23)	2.13 (1.82–2.49)	1.17 (1.03–1.33)	2.26 (1.96–2.61)	1.94 (1.65–2.29)
70–79	4.03 (3.79–4.28)	2.73 (2.33–3.19)	3.74 (3.33–4.20)	4.03 (3.43–4.73)	2.12 (1.86–2.42)	4.54 (3.93–5.25)	2.42 (2.01–2.91)
80+	11.0 (10.2–11.7)	4.40 (3.64–5.32)	6.06 (5.30–6.94)	5.78 (4.79–6.96)	4.07 (3.51–4.73)	7.17 (6.08–8.45)	19.0 (16.0–22.5)
**Sex**							
Women	1.27 (1.22–1.31)	1.17 (1.06–1.29)	1.34 (1.25–1.44)	1.32 (1.20–1.45)	1.30 (1.20–1.41)	1.04 (0.96–1.13)	0.87 (0.78–0.96)
**Immunosuppressive and chronic diseases**							
Severe immunosuppression[b]	0.97 (0.88–1.07)	0.94 (0.72–1.23)	1.01 (0.86–1.20)	1.08 (0.87–1.34)	0.98 (0.79–1.21)	0.89 (0.72–1.11)	0.70 (0.53–0.92)
Rheumatoid arthritis	1.17 (1.10–1.25)	0.96 (0.81–1.15)	1.09 (0.98–1.22)	1.16 (1.00–1.34)	1.20 (1.05–1.38)	1.10 (0.96–1.26)	1.13 (0.96–1.32)
Systemic lupus erythematosus	1.00 (0.58–1.74)	e	c	c	c	c	c
Inflammatory bowel disease	0.99 (0.84–1.17)	1.05 (0.69–1.61)	1.11 (0.84–1.48)	0.87 (0.57–1.33)	0.78 (0.52–1.17)	1.20 (0.85–1.69)	0.77 (0.46–1.29)
Chronic kidney disease	1.28 (1.07–1.52)	0.98 (0.58–1.65)	1.10 (0.80–1.52)	1.25 (0.84–1.88)	1.22 (0.83–1.80)	1.18 (0.81–1.72)	1.11 (0.72–1.70)
Asthma	0.90 (0.80–1.03)	1.00 (0.72–1.38)	0.91 (0.73–1.15)	1.02 (0.76–1.36)	0.99 (0.75–1.31)	0.87 (0.65–1.16)	0.93 (0.65–1.34)
Chronic obstructive pulmonary disease	1.01 (0.94–1.09)	0.69 (0.55–0.86)	0.97 (0.85–1.10)	0.97 (0.82–1.16)	1.06 (0.90–1.25)	1.00 (0.86–1.18)	1.00 (0.83–1.20)
Inhaled corticosteroids	1.14 (1.03–1.26)	1.13 (0.85–1.50)	1.22 (1.03–1.44)	1.39 (1.12–1.73)	1.00 (0.80–1.25)	1.19 (0.97–1.47)	0.74 (0.55–0.98)
Diabetes	1.35 (1.27–1.43)	1.18 (1.00–1.38)	1.18 (1.06–1.31)	1.22 (1.05–1.40)	1.41 (1.24–1.60)	1.25 (1.10–1.42)	1.09 (0.93–1.28)
Mood disorder	1.39 (1.29–1.50)	1.08 (0.87–1.34)	1.44 (1.26–1.63)	1.46 (1.23–1.73)	1.35 (1.15–1.58)	1.23 (1.04–1.46)	1.03 (0.84–1.27)
**Highest achieved education[c]**							
Short	N/A	1 (ref.)	1 (ref.)	1 (ref.)	1 (ref.)	1 (ref.)	NA
Intermediate	N/A	0.73 (0.65–0.81)	0.56 (0.52–0.60)	0.59 (0.54–0.66)	0.65 (0.59–0.71)	0.58 (0.53–0.64)	NA
High	N/A	0.59 (0.51–0.68)	0.37 (0.33–0.41)	0.36 (0.31–0.41)	0.47 (0.42–0.54)	0.42 (0.37–0.48)	NA
**Ethnicity**							
Danish	1 (ref.)	1 (ref.)	1 (ref.)	1 (ref.)	1 (ref.)	1 (ref.)	1 (ref.)
Other Western	1.79 (1.63–1.98)	0.94 (0.67–1.31)	1.07 (0.86–1.34)	0.86 (0.62–1.21)	1.09 (0.83–1.42)	0.90 (0.66–1.21)	10.7 (9.25–12.4)
Non-Western	4.16 (3.80–4.56)	2.46 (1.91–3.17)	2.21 (1.81–2.69)	2.84 (2.23–3.61)	1.52 (1.17–1.96)	3.37 (2.73–4.15)	15.1 (12.9–17.6)
**Smoking status**							
Never	N/A	N/A	1 (ref.)	1 (ref.)	1 (ref.)	1 (ref.)	1 (ref.)

Former	N/A	N/A	1.04 (0.96–1.12)	1.02 (0.92–1.13)	0.91 (0.82–1.00)	0.91 (0.82–1.00)	0.93 (0.83–1.04)
Current	N/A	N/A	1.11 (1.02–1.21)	0.98 (0.87–1.10)	1.00 (0.90–1.11)	1.25 (1.13–1.39)	1.09 (0.96–1.24)
**Alcohol consumption**[d]							
Low-risk	N/A	1 (ref.)	N/A	N/A	1 (ref.)	1 (ref.)	1 (ref.)
Intermediate-risk	N/A	0.84 (0.73–0.97)	N/A	N/A	0.83 (0.73–0.95)	0.75 (0.66–0.86)	0.87 (0.74–1.02)
High-risk	N/A	0.69 (0.57–0.83)	N/A	N/A	0.89 (0.77–1.03)	0.91 (0.79–1.05)	1.08 (0.91–1.27)
**Body mass index**			N/A				
Underweight	N/A	0.65 (0.41–1.01)	1.26 (1.01–1.57)	1.21 (0.90–1.62)	N/A	N/A	1.57 (1.18–2.10)
Normal	N/A	1 (ref.)	1 (ref.)	1 (ref.)	N/A	N/A	1 (ref.)
Overweight	N/A	1.16 (1.01–1.32)	1.08 (1.00–1.16)	0.94 (0.85–1.04)	N/A	N/A	0.93 (0.83–1.03)
Obese		1.03 (0.90–1.17)	1.16 (1.05–1.28)	0.98 (0.86–1.112	N/A	N/A	1.06 (0.92–1.23)
**Physical activity**							
Sedentary	N/A	1.03 (0.90–1.17)	1.17 (1.07–1.27)	1.20 (1.07–1.34)	N/A	N/A	2.00 (1.78–2.23)
Light	N/A	1 (ref.)	1 (ref.)	1 (ref.)	N/A	N/A	1 (ref.)
Moderate	N/A	1.08 (0.95–1.22)	0.92 (0.83–1.01)	0.93 (0.81–1.06)	N/A	N/A	0.95 (0.82–1.11)
Vigorous	N/A	1.40 (0.96–2.04)	1.66 (1.27–2.17)	1.45 (0.99–2.13)	N/A	N/A	0.88 (0.53–1.47)

Abbreviations: N/A, not applicable

[a]The overall model was not adjusted for variables with missing data, as that would predict outcome (missingness) perfectly.

[b]Variables for severe immunosuppression were combined because of low numbers.

[c]Categorized as short (<10 years), intermediate (10–15 years), and high education (>15 years), according to the United Nations Educational, Scientific and Cultural Organization's (UNESCO's) classification

[d]Categorized based on number of standardized alcohol units consumed weekly: low-risk (≤7 for women/≤14 for men) intermediate-risk (8–14 for women/15–21 for men); or high-risk (≥15 for women/≥22 for men).

[e]Not shown because of low numbers

Web Table 5. Cohort characteristics at follow-up start, by smoking status.[a]

	Smoking status					
	Never		Former		Current	
	n	%	n	%	n	%
**Age group (years)**						
40–49	13,045	36.5	7,187	23.3	7,151	32.0
50–59	8,830	24.7	8,013	26.0	6,860	30.7
60–69	8,222	23.0	8,595	27.9	5,534	24.8
70–79	3,929	11.0	4,861	15.8	2,095	9.4
80+	1,748	4.9	2,154	7.0	682	3.1
**Sex**						
Men	16,296	45.6	16,781	54.5	12,112	54.3
Women	19,477	54.4	14,028	45.5	10,211	45.7
**Immunosuppressive and chronic diseases**						
Rheumatoid arthritis	2,087	5.8	2,392	7.8	2,241	10.0
Systemic lupus erythematosus	25	0.1	35	0.1	30	0.1
Inflammatory bowel disease	385	1.1	416	1.4	261	1.2
Chronic kidney disease	171	0.5	259	0.8	101	0.5
Asthma	1,225	3.4	909	3.0	424	1.9
Chronic obstructive pulmonary disease	770	2.2	2,535	8.2	2,709	12.1
Inhaled corticosteroids	1,008	2.8	1,667	5.4	894	4.0
Diabetes	2,076	5.8	2,519	8.2	1,528	6.8
Mood disorder	1,407	3.9	1,499	4.9	1,825	8.2
Severe immunosuppression	797	2.2	1,082	3.5	612	2.7
**Highest achieved education**[b]						
Short	8,350	23.3	8,047	26.1	7,350	32.9
Intermediate	17,610	49.2	15,126	49.1	11,399	51.1
High	9,814	27.4	7,636	24.8	3,574	16.0
**Ethnicity**						
Danish	33,184	92.8	28,781	93.4	20,660	92.6
Other Western	1,111	3.1	1,262	4.1	853	3.8
Non-Western	1,478	4.1	767	2.5	809	3.6
**Alcohol consumption**[c]						
Low-risk	29,592	82.7	22,723	73.8	15,329	68.7
Intermediate-risk	4,177	11.7	4,726	15.3	3,197	14.3
High-risk	2,004	5.6	3,360	10.9	3,797	17.0
**Binge drinking weekly or more**						
No	33,883	94.7	28,123	91.3	18,628	83.4
Yes	1,891	5.3	2,686	8.7	3,695	16.6
**Body mass index category**						
Underweight	437	1.2	279	0.9	669	3.0
Normal	16,199	45.3	12,438	40.4	10,956	49.1
Overweight	13,468	37.6	12,866	41.8	7,715	34.6
Obese	5,669	15.8	5,226	17	2,981	13.4
**Physical activity**						
Sedentary	4,551	12.7	4,465	14.5	4,694	21.0
Light	22,101	61.8	19,319	62.7	13,704	61.4
Moderate	8,307	23.2	6,497	21.1	3,690	16.5
Vigorous	814	2.3	529	1.7	234	1.0

[a]We included the calibrated weights to statistically account for survey design and differential non-response in analyses. Thus, the numbers may not add up to the totals.
[b]Categorized as short (<10 years), intermediate (10–15 years), and high education (>15 years), according to the United Nations Educational, Scientific and Cultural Organization's (UNESCO's) classification
[c]Categorized based on number of standardized alcohol units consumed weekly: low-risk (≤7 for women/≤14 for men) intermediate-risk (8–14 for women/15–21 for men); or high-risk (≥15 for women/≥22 for men).

Web Table 6. Cohort characteristics at follow-up start, by alcohol consumption.[a]

	Alcohol consumption (units/week for females/males)						Binge drinking weekly or more			
	Low-risk (<7/14)		Intermediate-risk (8–14/15–21)		High-risk (≥15/22)		No		Yes	
	n	%	n	%	n	%	n	%	n	%
**Age group (years)**										
40–49	22,526	33.3	2,937	24.3	1,919	21.0	24,616	30.5	2,767	33.4
50–59	17,424	25.8	3,487	28.8	2,792	30.5	20,714	25.7	2,988	36.1
60–69	15,708	23.2	3,628	30.0	3,015	32.9	20,381	25.3	1,970	23.8
70–79	8,175	12.1	1,543	12.8	1,168	12.7	10,416	12.9	470	5.7
80+	3,813	5.6	504	4.2	267	2.9	4,507	5.6	76	0.9
**Sex**										
Men	34,372	50.8	4,950	40.9	5,867	64.0	38,679	48.0	6,510	78.7
Women	33,273	49.2	7,150	59.1	3,294	36.0	41,955	52.0	1,762	21.3
**Immunosuppressive and chronic diseases**										
Rheumatoid arthritis	4,973	7.4	797	6.6	951	10.4	5,883	7.3	838	10.1
Systemic lupus erythematosus	73	0.1	10	0.1	7	0.1	84	0.1	7	0.1
Inflammatory bowel disease	831	1.2	131	1.1	100	1.1	992	1.2	70	0.8
Chronic kidney disease	447	0.7	37	0.3	47	0.5	495	0.6	35	0.4
Asthma	2,038	3.0	329	2.7	191	2.1	2,356	2.9	202	2.4
Chronic obstructive pulmonary disease	4,281	6.3	708	5.9	1,025	11.2	5,242	6.5	771	9.3
Inhaled corticosteroids	2,758	4.1	418	3.5	393	4.3	3,290	4.1	278	3.4
Diabetes	4,850	7.2	591	4.9	682	7.4	5,505	6.8	618	7.5
Mood disorder	3,634	5.4	541	4.5	556	6.1	4,193	5.2	538	6.5
Severe immunosuppression	1,983	2.9	290	2.4	217	2.4	2,320	2.9	170	2.1
**Highest achieved education[b]**										
Short	19,024	28.1	2,508	20.7	2,215	24.2	21,727	26.9	2,020	24.4
Intermediate	33,465	49.5	5,913	48.9	4,758	51.9	39,677	49.2	4,458	53.9
High	15,156	22.4	3,679	30.4	2,188	23.9	19,230	23.8	1,794	21.7
**Ethnicity**										
Danish	62,425	92.3	11,534	95.3	8,667	94.6	74,830	92.8	7,795	94.2
Other Western	2,413	3.6	447	3.7	367	4.0	2,923	3.6	303	3.7
Non-Western	2,808	4.2	119	1.0	127	1.4	2,880	3.6	173	2.1
**Smoking status**										
Never	29,592	43.7	4,177	34.5	2,004	21.9	33,883	42.0	1,891	22.9
Former	22,723	33.6	4,726	39.1	3,360	36.7	28,123	34.9	2,686	32.5
Current	15,329	22.7	3,197	26.4	3,797	41.4	18,628	23.1	3,695	44.7
**Alcohol consumption[c]**										
Low-risk	-	-	-	-	-	-	66,059	81.9	1,586	19.2
Intermediate-risk	-	-	-	-	-	-	10,030	12.4	2,070	25.0
High-risk	-	-	-	-	-	-	4,545	5.6	4,616	55.8
**Binge drinking weekly or more**										
No	66,059	97.7	10,030	82.9	4,545	49.6	-	-	-	-
Yes	1,586	2.3	2,070	17.1	4,616	50.4	-	-	-	-
**Body mass index category**										

Underweight	1,004	1.5	218	1.8	164	1.8	1,295	1.6	90	1.1
Normal	29,602	43.8	6,108	50.5	3,883	42.4	36,543	45.3	3,051	36.9
Overweight	25,949	38.4	4,418	36.5	3,683	40.2	30,428	37.7	3,622	43.8
Obese	11,090	16.4	1,356	11.2	1,431	15.6	12,368	15.3	1,509	18.2
**Physical activity**										
Sedentary	10,663	15.8	1,334	11.0	1,713	18.7	12,172	15.1	1,537	18.6
Light	41,519	61.4	7,940	65.6	5,665	61.8	50,346	62.4	4,778	57.8
Moderate	14,152	20.9	2,656	22.0	1,686	18.4	16,707	20.7	1,787	21.6
Vigorous	1,312	1.9	169	1.4	97	1.1	1,408	1.7	170	2.1

[a]We included the calibrated weights to statistically account for survey design and differential non-response in analyses. Thus, the numbers may not add up to the totals.

[b]Categorized as short (<10 years), intermediate (10–15 years), and high education (>15 years), according to the United Nations Educational, Scientific and Cultural Organization's (UNESCO's) classification

[c]Categorized based on number of standardized alcohol units consumed weekly: low-risk (≤7 for women/≤14 for men) intermediate-risk (8–14 for women/15–21 for men); or high-risk (≥15 for women/≥22 for men).

Web Table 7. Cohort characteristics at follow-up start, by body mass index category.[a]

	Body mass index category							
	Underweight		Normal		Overweight		Obese	
	n	%	n	%	n	%	n	%
**Age group (years)**								
40–49	332	24.0	12,937	32.7	9,867	29.0	4,246	30.6
50–59	340	24.6	10,410	26.3	9,144	26.9	3,808	27.4
60–69	316	22.8	9,173	23.2	9,056	26.6	3,806	27.4
70–79	232	16.8	4,639	11.7	4,423	13.0	1,591	11.5
80+	164	11.8	2,434	6.1	1,560	4.6	426	3.1
**Sex**								
Men	262	18.9	16,713	42.2	20,890	61.4	7,324	52.8
Women	1,124	81.1	22,880	57.8	13,160	38.6	6,553	47.2
**Immunosuppressive and chronic diseases**								
Rheumatoid arthritis	159	11.5	2,462	6.2	2,570	7.5	1,528	11.0
Systemic lupus erythematosus	d	d	d	d	d	d	d	d
Inflammatory bowel disease	33	2.4	502	1.3	367	1.1	159	1.1
Chronic kidney disease	16	1.2	210	0.5	200	0.6	104	0.7
Asthma	31	2.3	996	2.5	994	2.9	537	3.9
Chronic obstructive pulmonary disease	255	18.4	2,416	6.1	2,083	6.1	1,260	9.1
Inhaled corticosteroids	123	8.9	1,411	3.6	1,288	3.8	746	5.4
Diabetes	61	4.4	1,435	3.6	2,379	7.0	2,247	16.2
Mood disorder	126	9.1	1,988	5.0	1,650	4.8	967	7.0
Severe immunosuppression	93	6.7	1,085	2.7	876	2.6	436	3.1
**Highest achieved education[b]**								
Short	504	36.4	9,232	23.3	9,235	27.1	4,776	34.4
Intermediate	554	40.0	19,001	48.0	17,587	51.7	6,994	50.4
High	328	23.7	11,360	28.7	7,228	21.2	2,107	15.2
**Ethnicity**								
Danish	1,297	93.6	36,784	92.9	31,601	92.8	12,943	93.3
Other Western	54	3.9	1,592	4.0	1,188	3.5	393	2.8
Non-Western	35	2.5	1,217	3.1	1,261	3.7	541	3.9
**Smoking status**								
Never	437	31.6	16,199	40.9	13,468	39.6	5,669	40.9
Former	279	20.2	12,438	31.4	12,866	37.8	5,226	37.7
Current	669	48.3	10,956	27.7	7,715	22.7	2,981	21.5
**Alcohol consumption[c]**								
Low-risk	1,004	72.4	29,602	74.8	25,949	76.2	11,090	79.9
Intermediate-risk	218	15.7	6,108	15.4	4,418	13.0	1,356	9.8
High-risk	164	11.8	3,883	9.8	3,683	10.8	1,431	10.3
**Binge drinking weekly or more**								
No	1,295	93.5	36,543	92.3	30,428	89.4	12,368	89.1
Yes	90	6.5	3,051	7.7	3,622	10.6	1,509	10.9
**Physical activity**								
Sedentary	359	25.9	4,559	11.5	5,062	14.9	3,730	26.9
Light	827	59.7	24,471	61.8	21,489	63.1	8,336	60.1
Moderate	176	12.7	9,630	24.3	6,974	20.5	1,714	12.4
Vigorous	23	1.7	933	2.4	525	1.5	97	0.7

[a]We included the calibrated weights to statistically account for survey design and differential non-response in analyses. Thus, the numbers may not add up to the totals.
[b]Categorized as short (<10 years), intermediate (10–15 years), and high education (>15 years), according to the United Nations Educational, Scientific and Cultural Organization's (UNESCO's) classification
[c]Categorized based on number of standardized alcohol units consumed weekly: low-risk (≤7 for women/≤14 for men) intermediate-risk (8–14 for women/15–21 for men); or high-risk (≥15 for women/≥22 for men).
[d]Not reported to preserve anonymity.

Web Table 8. Cohort characteristics at follow-up start, by physical activity.[a]

	Physical activity							
	Sedentary		Light		Moderate		Vigorous	
	n	%	n	%	n	%	n	%
**Age group (years)**								
40–49	3,724	27.2	14,965	27.1	7,770	42.0	924	58.6
50–59	3,328	24.3	15,048	27.3	4,932	26.7	395	25.0
60–69	2,761	20.1	15,342	27.8	4,066	22.0	183	11.6
70–79	2,042	14.9	7,331	13.3	1,448	7.8	65	4.1
80+	1,855	13.5	2,438	4.4	280	1.5	11	0.7
**Sex**								
Men	6,858	50.0	25,711	46.6	11,452	61.9	1,169	74.1
Women	6,852	50.0	29,413	53.4	7,043	38.1	409	25.9
**Immunosuppressive and chronic diseases**								
Rheumatoid arthritis	1,724	12.6	4,101	7.4	844	4.6	51	3.3
Systemic lupus erythematosus	d	d	d	d	d	d	d	d
Inflammatory bowel disease	208	1.5	659	1.2	181	1.0	14	0.9
Chronic kidney disease	206	1.5	279	0.5	41	0.2	5	0.3
Asthma	433	3.2	1,573	2.9	509	2.8	42	2.7
Chronic obstructive pulmonary disease	1,980	14.4	3,459	6.3	526	2.8	42	2.7
Inhaled corticosteroids	976	7.1	2,103	3.8	448	2.4	42	2.6
Diabetes	1,756	12.8	3,674	6.7	657	3.6	35	2.2
Mood disorder	1,267	9.2	2,760	5.0	636	3.4	68	4.3
Severe immunosuppression	716	5.2	1,490	2.7	268	1.5	17	1.1
**Highest achieved education[b]**								
Short	5,303	38.7	14,960	27.1	3,240	17.5	243	15.4
Intermediate	6,180	45.1	27,640	50.1	9,458	51.1	856	54.3
High	2,226	16.2	12,523	22.7	5,796	31.3	478	30.3
**Ethnicity**								
Danish	12,098	88.2	51,563	93.5	17,533	94.8	1,432	90.8
Other Western	565	4.1	1,990	3.6	611	3.3	60	3.8
Non-Western	1,047	7.6	1,571	2.9	351	1.9	85	5.4
**Smoking status**								
Never	4,551	33.2	22,101	40.1	8,307	44.9	814	51.6
Former	4,465	32.6	19,319	35.0	6,497	35.1	529	33.5
Current	4,694	34.2	13,704	24.9	3,690	20.0	234	14.8
**Alcohol consumption[c]**								
Low-risk	10,663	77.8	41,519	75.3	14,152	76.5	1,312	83.1
Intermediate-risk	1,334	9.7	7,940	14.4	2,656	14.4	169	10.7
High-risk	1,713	12.5	5,665	10.3	1,686	9.1	97	6.1
**Binge drinking weekly or more**								
No	12,172	88.8	50,346	91.3	16,707	90.3	1,408	89.2
Yes	1,537	11.2	4,778	8.7	1,787	9.7	170	10.8
**Body mass index category**								
Underweight	359	2.6	827	1.5	176	1.0	23	1.5
Normal	4,559	33.3	24,471	44.4	9,630	52.1	933	59.1
Overweight	5,062	36.9	21,489	39.0	6,974	37.7	525	33.3
Obese	3,730	27.2	8,336	15.1	1,714	9.3	97	6.1

[a]We included the calibrated weights to statistically account for survey design and differential non-response in analyses. Thus, the numbers may not add up to the totals.
[b]Categorized as short (<10 years), intermediate (10–15 years), and high education (>15 years), according to the United Nations Educational, Scientific and Cultural Organization's (UNESCO's) classification
[c]Categorized based on number of standardized alcohol units consumed weekly: low-risk (≤7 for women/≤14 for men) intermediate-risk (8–14 for women/15–21 for men); or high-risk (≥15 for women/≥22 for men).
[d]Not reported to preserve anonymity.

Web Table 9. Fully-adjusted hazard ratios (95% confidence intervals)[a] for the association between lifestyle factors and herpes zoster, by age and sex.

Lifestyle factor	Age group (years)					Sex	
	40–49	50–59	60–69	70–79	80+	Men	Women
**Smoking status**							
Never	(reference)	(reference)	(reference)	(reference)	(reference)	(reference)	(reference)
Former	1.20 (0.94-1.53)	1.15 (0.93-1.40)	1.21 (1.01-1.45)	1.00 (0.80-1.26)	1.11 (0.79-1.54)	1.08 (0.92-1.27)	1.18 (1.04-1.34)
Current	1.16 (0.89-1.51)	0.91 (0.71-1.16)	1.02 (0.82-1.27)	0.83 (0.61-1.12)	0.86 (0.50-1.48)	0.91 (0.74-1.11)	1.04 (0.89-1.21)
**Tobacco consumption (grams/day)[b]**					d		
1–5	1.76 (0.96-3.25)	1.07 (0.58-1.94)	0.89 (0.40-2.02)	0.81 (0.39-1.68)	d	0.55 (0.24-1.24)	1.31 (0.91-1.88)
6–10	1.12 (0.72-1.76)	1.12 (0.76-1.66)	1.37 (0.98-1.90)	1.16 (0.74-1.80)	d	1.16 (0.83-1.63)	1.18 (0.93-1.50)
11–15	0.84 (0.50-1.38)	0.91 (0.60-1.38)	0.92 (0.62-1.38)	0.52 (0.27-1.00)	d	0.90 (0.63-1.29)	0.84 (0.63-1.13)
16–20	1.15 (0.74-1.79)	0.78 (0.52-1.19)	1.10 (0.74-1.65)	0.59 (0.28-1.21)	d	0.89 (0.63-1.26)	1.01 (0.75-1.36)
21–25	1.08 (0.41-2.84)	0.33 (0.12-0.91)	0.86 (0.38-1.95)	0.91 (0.35-2.36)	d	0.78 (0.44-1.35)	0.72 (0.29-1.76)
26–30	1.14 (0.49-2.68)	d	d	d	d	0.72 (0.41-1.28)	0.70 (0.31-1.58)
30+	1.96 (0.77-5.04)	d	d	d	d	0.72 (0.36-1.46)	d
**Alcohol consumption[c]**							
Low-risk	(reference)	(reference)	(reference)	(reference)	(reference)	(reference)	(reference)
Intermediate-risk	1.10 (0.80-1.51)	0.92 (0.73-1.17)	0.94 (0.76-1.17)	0.99 (0.73-1.32)	0.68 (0.39-1.17)	0.83 (0.65-1.05)	1.02 (0.87-1.18)
High-risk	0.86 (0.55-1.32)	0.67 (0.48-0.94)	1.14 (0.89-1.44)	1.17 (0.87-1.57)	1.39 (0.73-2.64)	0.90 (0.73-1.11)	1.10 (0.89-1.36)
**Binge drinking weekly or more**							
No	(reference)	(reference)	(reference)	(reference)	(reference)	(reference)	(reference)
Yes	1.19 (0.83-1.70)	0.56 (0.38-0.81)	1.18 (0.88-1.58)	1.16 (0.72-1.89)	c	0.96 (0.77-1.20)	0.95 (0.70-1.30)
**Body mass index category**							
Underweight	1.58 (0.85-2.93)	0.60 (0.22-1.64)	0.90 (0.49-1.67)	0.88 (0.41-1.90)	1.03 (0.39-2.71)	1.42 (0.67-2.99)	0.90 (0.61-1.31)
Normal	(reference)	(reference)	(reference)	(reference)	(reference)	(reference)	(reference)
Overweight	1.13 (0.89-1.42)	1.01 (0.82-1.23)	0.87 (0.73-1.05)	1.01 (0.82-1.25)	0.99 (0.70-1.40)	1.01 (0.86-1.17)	0.98 (0.87-1.12)
Obese	1.04 (0.78-1.40)	1.05 (0.80-1.37)	0.99 (0.79-1.26)	0.89 (0.65-1.23)	0.74 (0.41-1.35)	1.05 (0.85-1.29)	0.99 (0.83-1.17)
**Physical activity**							
Sedentary	0.82 (0.59-1.14)	1.15 (0.88-1.51)	0.97 (0.75-1.25)	1.16 (0.90-1.51)	0.92 (0.67-1.28)	0.96 (0.79-1.18)	1.04 (0.88-1.22)
Light	(reference)	(reference)	(reference)	(reference)	(reference)	(reference)	(reference)
Moderate	0.83 (0.65-1.06)	1.06 (0.84-1.34)	1.11 (0.90-1.36)	1.10 (0.83-1.47)	0.62 (0.29-1.34)	0.99 (0.83-1.17)	0.99 (0.84-1.16)
Vigorous	1.03 (0.57-1.86)	1.09 (0.46-2.56)	1.03 (0.42-2.52)	d	d	0.75 (0.40-1.41)	1.77 (1.07-2.95)

[a]Computed using Cox regression with age as underlying time scale, stratified by birth cohort, and adjusted for sex, various immunosuppressive and chronic diseases (listed in Table 2 of main paper), ethnicity, and education level. Depending on exposure, we also adjusted for other lifestyle factors: smoking status was adjusted for weekly alcohol consumption, BMI and physical activity; weekly alcohol consumption and binge drinking was adjusted for smoking status, BMI and physical activity; and BMI and physical activity was adjusted for smoking status and weekly alcohol consumption. We included the calibrated weights to statistically account for survey design and differential non-response.

[b]Among daily smokers compared with never smokers.

[c]Categorized based on number of standardized alcohol units consumed weekly: low-risk (≤7 for women/≤14 for men) intermediate-risk (8–14 for women/15–21 for men); or high-risk (≥15 for women/≥22 for men).

[d]Not reported to preserve anonymity.

Web Table 10. Fully-adjusted hazard ratios (95% confidence intervals)[a] for the association between body mass index category and herpes zoster, by physical activity.

Body mass index	Physical activity			
	Sedentary	Light	Moderate	Vigorous
Underweight	1.56 (0.83-2.95)	0.65 (0.41-1.05)	1.57 (0.78- 3.16)	b
Normal	(reference)	(reference)	(reference)	(reference)
Overweight	1.05 (0.80-1.38)	0.98 (0.86-1.10)	1.05 (0.84-1.31)	b
Obese	1.03 (0.75-1.41)	1.00 (0.85-1.17)	1.05 (0.72-1.54)	b

[a]Computed using Cox regression with age as underlying time scale, stratified by birth cohort, and adjusted for sex, various immunosuppressive and chronic diseases, ethnicity, education level, smoking status and weekly alcohol consumption. We included the calibrated weights to statistically account for survey design and differential non-response.
[b]Not shown because of low numbers.

Web Table 11. Fully-adjusted hazard ratios (95% confidence intervals)[a] for the association between physical activity and herpes zoster, by body mass index category.

Physical activity	Body mass index category			
	**Underweight**	**Normal**	**Overweight**	**Obese**
Sedentary	2.18 (1.08-4.41)	0.95 (0.76-1.17)	0.98 (0.79-1.21)	1.03 (0.79-1.35)
Light	(reference)	(reference)	(reference)	(reference)
Moderate	1.99 (0.85-4.67)	0.95 (0.80-1.12)	1.02 (0.84-1.23)	0.99 (0.68-1.44)
Vigorous	b	1.33 (0.84-2.10)	0.55 (0.24-1.26)	b

[a]Computed using Cox regression with age as underlying time scale, stratified by birth cohort, and adjusted for sex, various immunosuppressive and chronic diseases, ethnicity, education level, smoking status and weekly alcohol consumption. We included the calibrated weights to statistically account for survey design and differential non-response.
[b]Not shown because of low numbers.

Web Table 12. Hazard ratios (95% confidence intervals) for the association between exposures and herpes zoster in model 4 from the main analysis and a sensitivity analysis adjusting additionally for working and civil status, limited to persons with complete data on all variables.[a]

Variable	Smoking status		Tobacco consumption		Alcohol consumption		Binge drinking		BMI category		Physical exercise	
	Main analysis[a]	Sensitivity analysis[b]	Main analysis[a]	Sensitivity analysis[b]	Main analysis[a]	Sensitivity analysis[b]	Main analysis[a]	Sensitivity analysis[b]	Main analysis[a]	Sensitivity analysis[b]	Main analysis[a]	Sensitivity analysis[b]
**Smoking status**												
Never	(reference)	(reference)	-	-	(reference)	(reference)	(reference)	(reference)	(reference)	(reference)	(reference)	(reference)
Former	1.18 (1.06-1.30)	1.17 (1.06-1.30)	-	-	1.18 (1.06-1.30)	1.17 (1.06-1.30)	1.18 (1.06-1.30)	1.17 (1.06-1.30)	1.18 (1.06-1.30)	1.17 (1.06-1.30)	1.18 (1.06-1.30)	1.17 (1.06-1.30)
Current	0.99 (0.88-1.13)	0.99 (0.88-1.13)	-	-	0.99 (0.88-1.13)	0.99 (0.88-1.13)	1.00 (0.88-1.13)	1.00 (0.88-1.13)	0.99 (0.88-1.13)	0.99 (0.88-1.13)	0.99 (0.88-1.12)	0.99 (0.87-1.12)
**Tobacco consumption (grams/day)[b]**												
1–5	-	-	0.98 (0.70-1.37)	0.98 (0.69-1.37)	-	-	-	-	-	-	-	-
6–10	-	-	1.18 (0.97-1.44)	1.18 (0.97-1.43)	-	-	-	-	-	-	-	-
11–15	-	-	0.88 (0.69-1.11)	0.87 (0.69-1.10)	-	-	-	-	-	-	-	-
16–20	-	-	0.98 (0.78-1.23)	0.97 (0.77-1.22)	-	-	-	-	-	-	-	-
21–25	-	-	0.79 (0.49-1.27)	0.78 (0.48-1.25)	-	-	-	-	-	-	-	-
26–30	-	-	0.77 (0.48-1.23)	0.76 (0.47-1.21)	-	-	-	-	-	-	-	-
30+	-	-	0.87 (0.47-1.60)	0.86 (0.47-1.58)	-	-	-	-	-	-	-	-
**Alcohol consumption[c]**												
Low-risk	(reference)	(reference)	(reference)	(reference)	(reference)	(reference)	-	-	(reference)	(reference)	(reference)	(reference)
Intermediate-risk	0.95 (0.83-1.07)	0.94 (0.83-1.07)	0.84 (0.70-1.01)	0.84 (0.70-1.01)	0.95 (0.83-1.07)	0.94 (0.83-1.07)	-	-	0.95 (0.83-1.07)	0.94 (0.83-1.07)	0.94 (0.83-1.07)	0.94 (0.83-1.07)
High-risk	1.01 (0.87-1.17)	1.01 (0.87-1.17)	0.91 (0.74-1.14)	0.91 (0.73-1.13)	1.01 (0.87-1.17)	1.01 (0.87-1.17)	-	-	1.01 (0.87-1.17)	1.01 (0.87-1.17)	1.01 (0.87-1.17)	1.01 (0.87-1.17)
**Binge drinking weekly or more**												
No	-	-	-	-	-	-	(reference)	(reference)	-	-	-	-
Yes	-	-	-	-	-	-	0.95 (0.79-1.13)	0.95 (0.79-1.14)	-	-	-	-
**Body mass index category**												
Underweight	1.00 (0.71-1.41)	0.99 (0.70-1.40)	1.13 (0.78-1.65)	1.13 (0.77-1.65)	1.00 (0.71-1.41)	0.99 (0.70-1.40)	1.00 (0.71-1.41)	0.99 (0.70-1.40)	1.00 (0.71-1.41)	0.99 (0.70-1.41)	-	-
Normal	(reference)	(reference)	(reference)	(reference)	(reference)	(reference)	(reference)	(reference)	(reference)	(reference)	-	-
Overweight	0.99 (0.89-1.09)	0.99 (0.89-1.09)	1.01 (0.88-1.16)	1.01 (0.88-1.16)	0.99 (0.89-1.09)	0.99 (0.89-1.09)	0.99 (0.90-1.09)	0.99 (0.89-1.09)	0.99 (0.89-1.09)	0.99 (0.89-1.09)	-	-

Obese	1.03 (0.90-1.17)	1.02 (0.89-1.17)	1.04 (0.87-1.25)	1.03 (0.86-1.24)	1.03 (0.90-1.17)	1.02 (0.89-1.17)	1.03 (0.90-1.18)	1.03 (0.90-1.17)	1.03 (0.90-1.17)	1.02 (0.90-1.17)	-	-
**Physical activity**												
Sedentary	1.00 (0.88-1.14)	1.00 (0.88-1.14)	1.13 (0.95-1.33)	1.12 (0.95-1.33)	1.00 (0.88-1.14)	1.00 (0.88-1.14)	1.00 (0.88-1.14)	1.00 (0.88-1.14)	-	-	1.01 (0.88-1.14)	1.00 (0.88-1.14)
Light	(reference)	(reference)	(reference)	(reference)	(reference)	(reference)	(reference)	(reference)	-	-	(reference)	(reference)
Moderate	0.97 (0.86-1.10)	0.98 (0.87-1.10)	1.01 (0.85-1.18)	1.01 (0.86-1.19)	0.97 (0.86-1.10)	0.98 (0.87-1.10)	0.97 (0.86-1.10)	0.98 (0.87-1.10)	-	-	0.97 (0.86-1.10)	0.98 (0.87-1.10)
Vigorous	1.08 (0.72-1.62)	1.09 (0.72-1.64)	1.11 (0.64-1.95)	1.11 (0.64-1.95)	1.08 (0.72-1.62)	1.09 (0.72-1.64)	1.08 (0.72-1.62)	1.09 (0.72-1.64)	-	-	1.08 (0.72-1.62)	1.09 (0.72-1.63)
**Sex**												
Men	(reference)	(reference)	(reference)	(reference)	(reference)	(reference)	(reference)	(reference)	(reference)	(reference)	(reference)	(reference)
Women	1.45 (1.32-1.60)	1.45 (1.32-1.60)	1.44 (1.26-1.64)	1.42 (1.25-1.63)	1.45 (1.32-1.60)	1.45 (1.32-1.60)	1.44 (1.31-1.58)	1.44 (1.31-1.59)	1.45 (1.32-1.60)	1.45 (1.32-1.60)	1.45 (1.32-1.60)	1.45 (1.32-1.60)
**Immunosuppressive and chronic diseases**												
Rheumatoid arthritis	1.03 (0.88-1.22)	1.03 (0.87-1.22)	0.90 (0.72-1.14)	0.89 (0.71-1.13)	1.03 (0.88-1.22)	1.03 (0.87-1.21)	1.04 (0.88-1.22)	1.03 (0.87-1.21)	1.03 (0.88-1.22)	1.03 (0.87-1.21)	1.04 (0.88-1.22)	1.03 (0.87-1.21)
Systemic lupus erythematosus	1.55 (0.65-3.72)	1.53 (0.64-3.67)	0.85 (0.19-3.83)	0.84 (0.18-3.81)	1.55 (0.65-3.72)	1.53 (0.64-3.67)	1.56 (0.65-3.73)	1.54 (0.64-3.68)	1.55 (0.65-3.73)	1.53 (0.64-3.67)	1.55 (0.65-3.71)	1.53 (0.64-3.66)
Inflammatory bowel disease	1.23 (0.86-1.75)	1.22 (0.85-1.74)	1.18 (0.73-1.89)	1.16 (0.72-1.87)	1.23 (0.86-1.75)	1.22 (0.85-1.74)	1.23 (0.86-1.75)	1.22 (0.85-1.74)	1.23 (0.86-1.75)	1.22 (0.85-1.74)	1.23 (0.86-1.75)	1.22 (0.85-1.74)
Chronic obstructive pulmonary disease	1.23 (1.02-1.49)	1.23 (1.01-1.48)	1.33 (1.02-1.73)	1.32 (1.01-1.71)	1.23 (1.02-1.49)	1.23 (1.01-1.48)	1.23 (1.02-1.49)	1.23 (1.02-1.48)	1.23 (1.02-1.49)	1.23 (1.02-1.48)	1.23 (1.02-1.49)	1.23 (1.02-1.48)
Asthma	1.11 (0.83-1.51)	1.11 (0.82-1.50)	1.07 (0.69-1.67)	1.07 (0.69-1.66)	1.11 (0.83-1.51)	1.11 (0.82-1.50)	1.12 (0.83-1.51)	1.11 (0.82-1.50)	1.12 (0.83-1.51)	1.11 (0.82-1.50)	1.12 (0.83-1.51)	1.11 (0.82-1.50)
Chronic kidney disease	1.50 (0.96-2.33)	1.49 (0.96-2.32)	1.61 (0.86-3.00)	1.59 (0.85-2.98)	1.50 (0.96-2.33)	1.49 (0.96-2.32)	1.50 (0.96-2.34)	1.49 (0.96-2.32)	1.50 (0.97-2.34)	1.49 (0.96-2.32)	1.50 (0.96-2.33)	1.49 (0.96-2.32)
Mood disorder	1.18 (0.97-1.43)	1.16 (0.95-1.41)	1.13 (0.88-1.45)	1.10 (0.85-1.41)	1.18 (0.97-1.43)	1.16 (0.95-1.41)	1.18 (0.97-1.43)	1.16 (0.96-1.41)	1.18 (0.97-1.43)	1.16 (0.95-1.41)	1.18 (0.97-1.43)	1.16 (0.95-1.41)
Diabetes	1.01 (0.85-1.20)	1.01 (0.85-1.20)	1.17 (0.92-1.48)	1.16 (0.92-1.47)	1.01 (0.85-1.20)	1.01 (0.85-1.20)	1.01 (0.85-1.20)	1.01 (0.85-1.20)	1.01 (0.85-1.20)	1.01 (0.85-1.20)	1.02 (0.86-1.20)	1.01 (0.85-1.20)
Inhaled corticosteroids	1.02 (0.79-1.31)	1.02 (0.79-1.31)	1.10 (0.76-1.59)	1.10 (0.77-1.59)	1.02 (0.79-1.31)	1.02 (0.79-1.31)	1.02 (0.79-1.31)	1.02 (0.79-1.31)	1.02 (0.79-1.31)	1.02 (0.79-1.31)	1.02 (0.79-1.31)	1.02 (0.79-1.31)
HIV	c	c	c	c	c	c	C	c	C	C	c	C
Other immunosuppressive disease	c	c	c	c	c	c	C	c	C	C	c	C
Leukemia	2.37 (1.32-4.24)	2.37 (1.33-4.23)	4.57 (2.36-8.83)	4.45 (2.35-8.80)	2.37 (1.32-4.24)	2.37 (1.33-4.23)	2.7 (1.32-4.24)	2.37 (1.33-4.24)	2.36 (1.32-4.23)	2.36 (1.32-4.22)	2.37 (1.32-4.24)	2.37 (1.33-4.23)
Lymphoma	2.16 (1.40-3.33)	2.16 (1.40-3.32)	1.83 (0.91-3.66)	1.83 (0.91-3.65)	2.16 (1.40-3.33)	2.16 (1.40-3.32)	2.16 (1.40-3.32)	2.15 (1.40-3.31)	2.16 (1.41-3.33)	2.16 (1.40-3.32)	2.16 (1.40-3.32)	2.16 (1.40-3.31)
Myeloma	3.03 (1.40-6.58)	2.99 (1.38-6.50)	1.70 (0.31-9.36)	1.68 (0.30-9.923)	3.03 (1.40-6.58)	2.99 (1.38-6.50)	3.04 (1.40-6.59)	3.00 (1.38-6.51)	3.02 (1.39-6.54)	2.98 (1.37-6.46)	3.03 (1.40-6.57)	2.99 (1.38-6.49)
Oral corticosteroids	1.36 (1.01-1.83)	1.36 (1.01-1.82)	1.38 (0.92-2.07)	1.38 (0.92-2.07)	1.36 (1.01-1.83)	1.36 (1.01-1.82)	1.36 (1.01-1.83)	1.36 (1.01-1.83)	1.36 (1.01-1.83)	1.36 (1.01-1.83)	1.36 (1.01-1.83)	1.36 (1.01-1.82)
Hematopoietic stem cell transplant	1.46 (0.55-3.83)	1.43 (0.55-3.74)	0.28 (0.04-2.14)	0.28 (0.04-2.12)	1.46 (0.55-3.83)	1.43 (0.55-3.74)	1.45 (0.55-3.82)	1.43 (0.54-3.74)	1.46 (0.56-3.84)	1.43 (0.55-3.74)	1.46 (0.55-3.82)	1.43 (0.55-3.73)

Other immunosuppressants	1.67 (1.23-2.26)	1.65 (1.22-2.24)	1.88 (1.22-2.90)	1.87 (1.22-2.88)	1.67 (1.23-2.26)	1.65 (1.22-2.24)	1.66 (1.23-2.26)	1.65 (1.22-2.24)	1.67 (1.23-2.26)	1.66 (1.22-2.24)	1.67 (1.23-2.26)	1.66 (1.22-2.24)
**Highest achieved education[d]**												
Short	(reference)	(reference)	(reference)	(reference)	(reference)	(reference)	(reference)	(reference)	(reference)	(reference)	(reference)	(reference)
Intermediate	0.96 (0.86-1.07)	0.96 (0.87-1.07)	0.98 (0.85-1.13)	0.99 (0.86-1.14)	0.96 (0.87-1.07)	0.96 (0.87-1.07)	0.96 (0.86-1.07)	0.96 (0.87-1.07)	0.96 (0.86-1.07)	0.96 (0.87-1.07)	0.96 (0.86-1.07)	0.96 (0.87-1.07)
High	1.01 (0.89-1.15)	1.02 (0.90-1.16)	0.93 (0.78-1.11)	0.95 (0.80-1.13)	1.02 (0.90-1.16)	1.02 (0.90-1.16)	1.01 (0.89-1.14)	1.02 (0.90-1.16)	1.01 (0.89-1.15)	1.02 (0.90-1.16)	1.01 (0.89-1.14)	1.02 (0.90-1.16)
**Ethnicity**												
Danish	(reference)	(reference)	(reference)	(reference)	(reference)	(reference)	(reference)	(reference)	(reference)	(reference)	(reference)	(reference)
Other Western	1.16 (0.91-1.50)	1.17 (0.91-1.50)	1.29 (0.93-1.79)	1.29 (0.92-1.79)	1.16 (0.91-1.50)	1.17 (0.91-1.50)	1.16 (0.91-1.50)	1.17 (0.91-1.50)	1.16 (0.91-1.50)	1.17 (0.91-1.50)	1.16 (0.90-1.49)	1.16 (0.91-1.50)
Non-Western	0.88 (0.61-1.27)	0.86 (0.59-1.24)	0.75 (0.46-1.20)	0.72 (0.45-1.16)	0.88 (0.61-1.27)	0.86 (0.59-1.24)	0.898 (0.61-1.27)	0.86 (0.59-1.24)	0.88 (0.61-1.27)	0.86 (0.60-1.24)	0.88 (0.61-1.27)	0.85 (0.59-1.23)
**Working status**												
Not working	-	(reference)	-	(reference)	-	(reference)	-	(reference)	-	(reference)	-	(reference)
Working	-	0.88 (0.77-1.02)	-	0.86 (0.72-1.04)	-	0.88 (0.77-1.02)	-	0.88 (0.77-1.02)	-	0.88 (0.77-1.02)	-	0.88 (0.77-1.02)
**Civil status**												
Single	-	(reference)	-	(reference)	-	(reference)	-	(reference)	-	(reference)	-	(reference)
Cohabitating	-	1.05 (0.95-1.17)	-	1.00 (0.87-1.15)	-	1.05 (0.95-1.17)	-	1.05 (0.94-1.17)	-	1.05 (0.95-1.17)	-	1.05 (0.95-1.17)
**Observations**	99141	99141	61111	61111	99141	99141	99141	99141	99141	99141	99141	99141

[a]Computed using Cox regression with age as underlying time scale, stratified by birth cohort, and adjusted for sex, various immunosuppressive and chronic diseases (listed in Table 2 of main paper), ethnicity, and education level. Depending on exposure, we also adjusted for other lifestyle factors: smoking status was adjusted for weekly alcohol consumption, BMI and physical activity; weekly alcohol consumption and binge drinking was adjusted for smoking status, BMI and physical activity; and BMI and physical activity was adjusted for smoking status and weekly alcohol consumption. We included the calibrated weights to statistically account for survey design and differential non-response.

[b]Adjusting additionally for working status and civil status.

[c]Categorized based on number of standardized alcohol units consumed weekly: low-risk (≤7 for women/≤14 for men) intermediate-risk (8–14 for women/15–21 for men); or high-risk (≥15 for women/≥22 for men).

[d]Categorized as short (<10 years), intermediate (10–15 years), and high education (>15 years), according to the United Nations Educational, Scientific and Cultural Organization's (UNESCO's) classification

Web Table 13. Hazard ratios (95% confidence interval) for the association between lifestyle and anthropometric factors and herpes Zoster, 2010 Danish National Health Survey, main analysis and sensitivity analysis adjusting additionally for the Charlson Comorbidity Index (CCI).

**Lifestyle and anthropometric factors**	**Main analysis[a]**	**Sensitivity analysis, adjusting for CCI categories[b]**	**Sensitivity analysis, adjusting for individual diseases of the CCI[c]**
Smoking status			
Never	(Referent)	(Referent)	(Referent)
Former	1.17 (1.06,1.30)	1.17 (1.06,1.29)	1.17 (1.06,1.30)
Current	1.00 (0.89,1.13)	0.99 (0.88,1.12)	1.00 (0.89,1.13)
Tobacco consumption (grams/day)[d]			
1–5	1.09 (0.78,1.51)	1.08 (0.78,1.50)	1.08 (0.78,1.50)
6–10	1.19 (0.98,1.44)	1.17 (0.97,1.42)	1.17 (0.97,1.43)
11–15	0.88 (0.70,1.11)	0.87 (0.69,1.09)	0.87 (0.69,1.10)
16–20	0.98 (0.78,1.23)	0.96 (0.77,1.20)	0.96 (0.77,1.21)
21–25	0.77 (0.48,1.24)	0.75 (0.47,1.21)	0.76 (0.47,1.21)
26–30	0.74 (0.46,1.18)	0.73 (0.46,1.16)	0.73 (0.46,1.16)
30+	0.84 (0.46,1.54)	0.82 (0.45,1.51)	0.81 (0.44,1.50)
Alcohol consumption[e]			
Low-risk	(Referent)	(Referent)	(Referent)
Intermediate-risk	0.95 (0.84,1.07)	0.95 (0.84,1.08)	0.95 (0.84,1.08)
High-risk	0.99 (0.85,1.15)	0.99 (0.85,1.14)	0.99 (0.85,1.14)
Binge drinking weekly or more			
No	(Referent)	(Referent)	(Referent)
Yes	0.93 (0.77,1.11)	0.93 (0.77,1.11)	0.93 (0.77,1.11)
Body mass index category			
Underweight	0.96 (0.69, 1.36)	0.96 (0.68, 1.36)	0.97 (0.69, 1.37)
Normal	(Referent)	(Referent)	(Referent)
Overweight	0.99 (0.90,1.09)	0.99 (0.90,1.09)	0.99 (0.90,1.09)
Obese	1.01 (0.88, 1.15)	1.00 (0.88, 1.14)	1.00 (0.88, 1.15)
Physical activity			
Sedentary	0.99 (0.87, 1.13)	0.98 (0.86, 1.12)	0.99 (0.87, 1.12)
Light	(Referent)	(Referent)	(Referent)

Moderate	0.98 (0.88,1.11)	0.99 (0.88,1.11)	0.99 (0.88,1.11)
Vigorous	1.08 (0.72, 1.61)	1.08 (0.73, 1.62)	1.09 (0.73, 1.62)

[a]Computed using Cox regression with age as underlying time scale, stratified by birth cohort, and adjusted for sex, various immunosuppressive and chronic diseases (listed in Table 2 of main paper), ethnicity, and education level. Depending on exposure, we also adjusted for other lifestyle factors: smoking status was adjusted for weekly alcohol consumption, BMI and physical activity; weekly alcohol consumption and binge drinking was adjusted for smoking status, BMI and physical activity; and BMI and physical activity was adjusted for smoking status and weekly alcohol consumption. We included the calibrated weights to statistically account for survey design and differential non-response.

[b]Adjusted for variables in main analysis plus Charlson Comorbidity Index category (0, 1, 2, or 3 or more) based on new diagnoses in the year before baseline.

[c]Adjusted for variables in main analysis plus individual diseases in the Charlson Comorbidity Index based on new diagnoses in the year before baseline.

[d]Among daily smokers compared with never smokers.

[e]Categorized based on number of standardized alcohol units consumed weekly: low-risk (≤7 for women/≤14 for men) intermediate-risk (8–14 for women/15–21 for men); or high-risk (≥15 for women/≥22 for men).
